# Selection-Driven Accumulation of Suppressor Mutants in *Bacillus subtilis*: The Apparent High Mutation Frequency of the Cryptic *gudB* Gene and the Rapid Clonal Expansion of *gudB^+^* Suppressors Are Due to Growth under Selection

**DOI:** 10.1371/journal.pone.0066120

**Published:** 2013-06-13

**Authors:** Katrin Gunka, Lorena Stannek, Rachel A. Care, Fabian M. Commichau

**Affiliations:** Department of General Microbiology, Georg-August-University Göttingen, Göttingen, Germany; University of Groningen, Groningen Institute for Biomolecular Sciences and Biotechnology, The Netherlands

## Abstract

Soil bacteria like *Bacillus subtilis* can cope with many growth conditions by adjusting gene expression and metabolic pathways. Alternatively, bacteria can spontaneously accumulate beneficial mutations or shape their genomes in response to stress. Recently, it has been observed that a *B. subtilis* mutant lacking the catabolically active glutamate dehydrogenase (GDH), RocG, mutates the cryptic *gudB^CR^* gene at a high frequency. The suppressor mutants express the active GDH GudB, which can fully replace the function of RocG. Interestingly, the cryptic *gudB^CR^* allele is stably inherited as long as the bacteria synthesize the functional GDH RocG. Competition experiments revealed that the presence of the cryptic *gudB^CR^* allele provides the bacteria with a selective growth advantage when glutamate is scarce. Moreover, the lack of exogenous glutamate is the driving force for the selection of mutants that have inactivated the active *gudB* gene. In contrast, two functional GDHs are beneficial for the cells when glutamate was available. Thus, the amount of GDH activity strongly affects fitness of the bacteria depending on the availability of exogenous glutamate. At a first glance the high mutation frequency of the cryptic *gudB^CR^* allele might be attributed to stress-induced adaptive mutagenesis. However, other loci on the chromosome that could be potentially mutated during growth under the selective pressure that is exerted on a GDH-deficient mutant remained unaffected. Moreover, we show that a GDH-proficient *B. subtilis* strain has a strong selective growth advantage in a glutamate-dependent manner. Thus, the emergence and rapid clonal expansion of the active *gudB* allele can be in fact explained by spontaneous mutation and growth under selection without an increase of the mutation rate. Moreover, this study shows that the selective pressure that is exerted on a maladapted bacterium strongly affects the apparent mutation frequency of mutational hot spots.

## Introduction

The high abundance of glutamate in many living organisms suggests that this metabolite fulfils fundamental tasks in the cell [1]–[4]. Indeed, glutamate delivers the majority of amino groups for biosynthesis of nitrogen-containing building blocks [5], [6]. Moreover, beside its important role in anabolism, glutamate serves as an osmoprotectant in some archaea and bacteria [7], [8]. The Gram-positive model organism *Bacillus subtilis*, however, needs glutamate in high amounts to synthesize proline, which serves as a compatible solute to protect cells growing under high external osmotic pressure [9].

**Figure 1:**
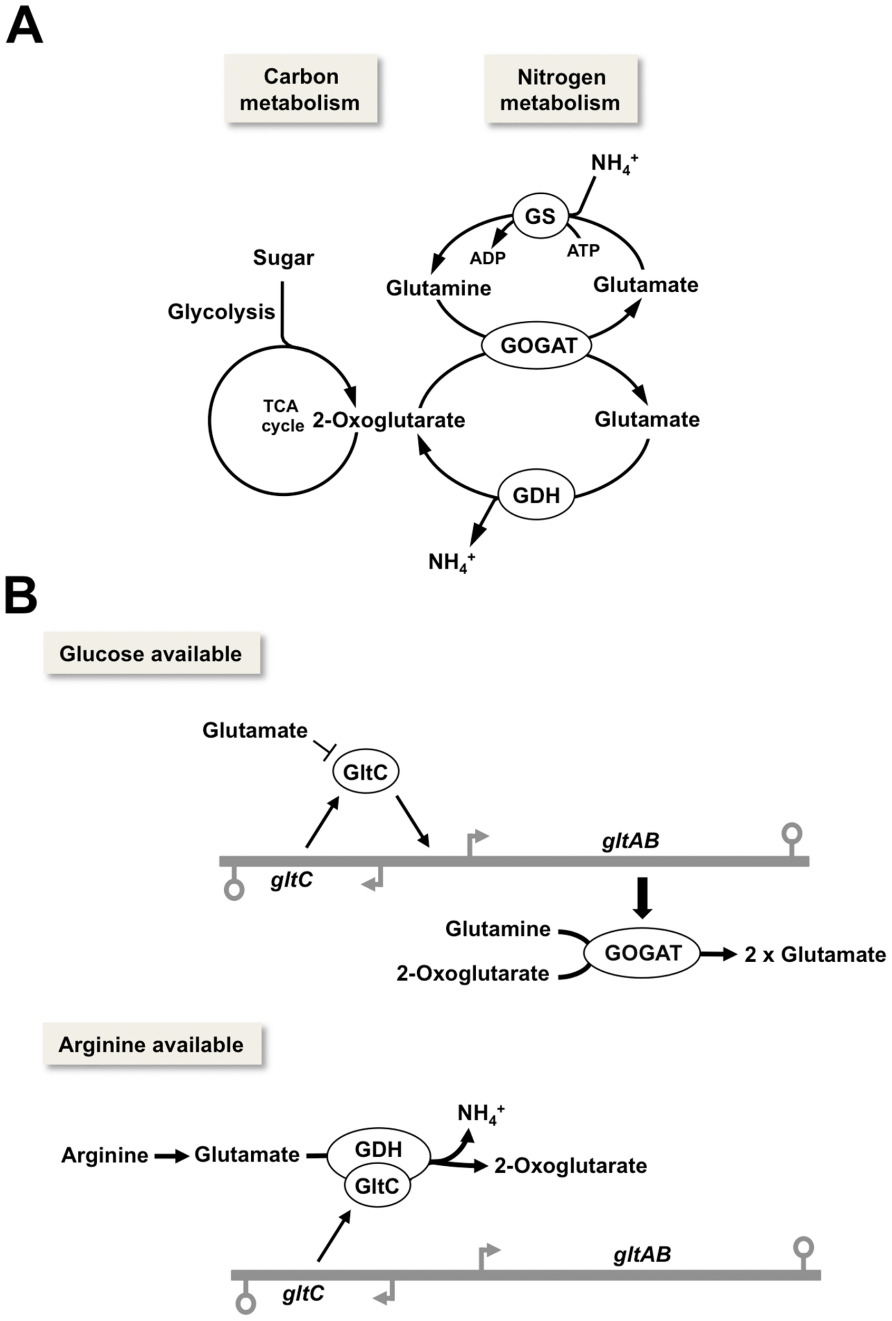
Glutamate biosynthesis and degradation in *B. subtilis*. (A) The link between carbon and nitrogen metabolism. GS, glutamine synthetase; GOGAT, glutamate synthase; GDH, glutamate dehydrogenase. (B) In the presence of glucose GltC activates the *gltAB* operon and the synthesized GOGAT converts 2-oxoglutarate and glutamine to glutamate. In the presence of arginine the GDH RocG is synthesized and the catabolically active enzyme binds to GltC and inhibits its DNA-binding activity.

In *B. subtilis* glutamate is exclusively synthesized by the combined action of the glutamine synthetase (GS) and the glutamate synthase (GOGAT) that are encoded by the *glnA* and *gltAB* genes, respectively (for a recent review [10]). The glutamate dehydrogenase (GDH) RocG, which is encoded by the *rocG* gene, strictly degrades glutamate *in vivo* (Figure 1A; [11]). The inability of RocG to synthesize glutamate in the background of a *B. subtilis* cell is caused by the very low affinity of the enzyme for ammonium [12], [13].

As glutamate synthesis and degradation link carbon to nitrogen metabolism, this important metabolic intersection has to be tightly controlled. Indeed, in *B. subtilis* and in many other organisms, glutamate biosynthesis and degradation are subject to dual control by signals derived from carbon and nitrogen [14]–[16]. During growth of *B. subtilis* cells in the presence of their preferred carbon source glucose and ammonium as the single source of nitrogen, the transcription factor GltC activates the expression of the *gltAB* genes and the encoded GOGAT synthesizes glutamate (Figure 1B; [17], [18]). At the same time, transcription of the *rocG* gene encoding the catabolically active GDH, RocG is strongly inhibited by the pleiotropic transcription factor CcpA [19]. This carbon source-dependent transcriptional activation and inhibition of the *gltAB* and *rocG* genes, respectively, allows the bacteria to produce glutamate, which is needed in high amounts to achieve high growth rates when external glutamate is scarce. If glutamate is provided to the cell, the slight inhibition of the transcription activator GltC results in a twofold reduced expression of the *gltAB* genes, and exogenously provided together with endogenously formed glutamate is incorporated into biomass [20], [21]. In the presence of arginine or related amino acids such as ornithine, which can be degraded to glutamate, the GDH-encoding *rocG* gene is strongly induced [11], [19]. This has two implications for the cells. First, the bifunctional GDH RocG directly binds to and prevents GltC from transcription activation of the *gltAB* genes, encoding the glutamate-synthesizing GOGAT (Figure 1B; [22], [23]). Second, the catabolically active GDH enables the bacteria to utilize glutamate as an additional carbon source that is fed into the tricarboxylic acid cycle (see Figure 1A). This elegant regulatory mechanism allows the bacteria to accurately adjust glutamate metabolism depending on the available carbon and nitrogen sources.

The genome of the *B. subtilis* laboratory strain 168, which is used worldwide in basic research and industry, contains two GDH-encoding genes, *rocG* and *gudB^CR^* [24]. However, only the *rocG* gene encodes a functional GDH, whereas the *gudB^CR^* gene is cryptic and encodes the enzymatically inactive GDH, GudB^CR^ (formerly designated as GudB, [11], [25]). The GDH GudB^CR^ is enzymatically inactive and extremely unstable because it contains a duplication of three amino acids in its active centre [11], [26], [27]. The duplication of these amino acids in GudB^CR^ is due to a perfect 9 bp-long direct repeat (DR) that is present in the cryptic *gudB^CR^* gene. In contrast to the laboratory strain 168, the genomes of closely related “wild” wild-type *B. subtilis* strains such as ATCC 6051 and NCIB 3610 encode two functional GDHs, RocG and GudB [25]. It is tempting to speculate that the *gudB^+^* gene became cryptic during domestication of a *B. subtilis* “wild” wild-type strain [25], [28].

We are interested in the control of glutamate homeostasis in *B.* *subtilis*. As described above, due to its outstanding role in cellular metabolism, the intracellular pool of glutamate has to be tightly adjusted for cellular vitality. Indeed, disruption of the arginine-degradative pathway by inactivation of the *rocG* gene causes a severe growth defect of the bacteria on rich medium [11], [27]. Although the reason for the growth defect remains to be elucidated, glutamate homeostasis is obviously completely out of balance because the lack of GDH activity causes a block in glutamate catabolism and results in the overexpression of the *gltAB* genes, encoding the glutamate-synthesizing GOGAT [20], [29]. Interestingly, the growth defect of a *rocG* mutant is suppressed by the emergence of mutants that have activated the cryptic *gudB^CR^* gene by the precise deletion of one part of the perfect DR repeat that is present in the gene [11], [27]. The decryptification of the *gudB^CR^* gene occurs at a very high frequency of 10^−4^ and the resulting *gudB* suppressor mutants synthesize the enzymatically and regulatory active GDH GudB, which may control as RocG the activity of the transcription factor GltC (see above; [22], [27]).

In this work we addressed the question of how GDH activity affects fitness of the bacteria. Moreover, we show that the availability of glutamate is the driving force for the selection of mutants expressing the active *gudB^+^* and inactive *gudB^CR^* alleles, respectively. Suppressor mutants that have decryptified the *gudB^CR^* gene and synthesize the enzymatically active GDH, GudB have an extremely strong growth advantage over cells lacking a functional GDH. Thus, the rapid emergence and clonal expansion of the active *gudB^+^* allele in a population of cells can be explained rather by spontaneous mutation than by adaptive mutagenesis.

## Results

### GDH Activity Determines Fitness of *B. subtilis* Depending on the Availability of Glutamate

**Figure 2:**
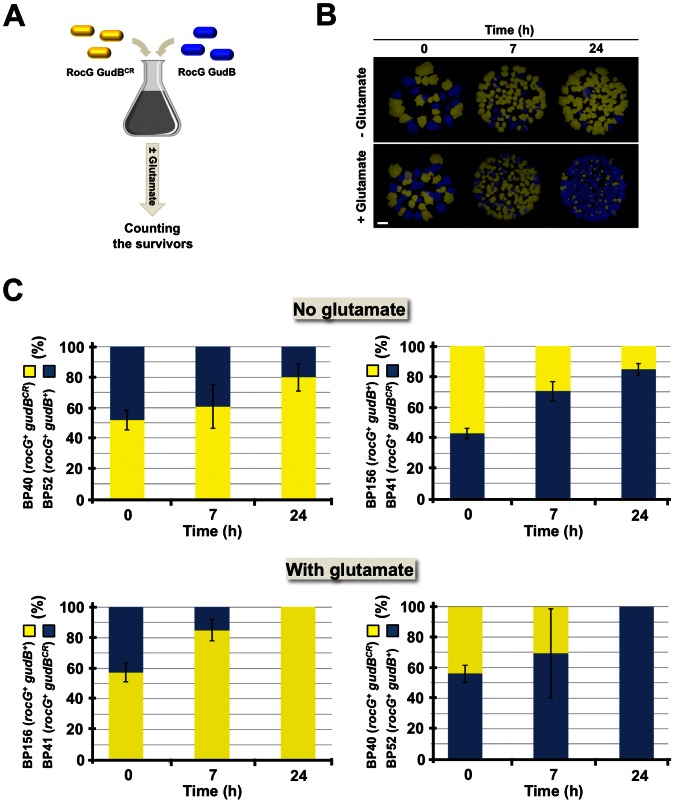
Intraspecies competition experiment to identify the selective advantage for keeping the *gudB^CR^* allele in the laboratory strain 168. (A) Mixed populations of strains BP40 (*rocG^+^ gudB^CR^ amyE::yfp*) and BP52 (*rocG^+^ gudB^+^ amyE::cfp*) or BP41 (*rocG^+^ gudB^CR^ amyE::cfp*) and BP156 (*rocG^+^ gudB^+^ amyE::yfp*) were grown for up to 24 h in C minimal medium supplemented with glucose and ammonium, and in minimal medium supplemented with glucose, ammonium and glutamate. (B) Prior to co-cultivation (0 h), and after 7 h and 24 h of growth dilutions of cells were plated on complex medium. The surviving cells that emerged after 12 h of incubation were identified by fluorescence microscopy and counted. Exposure time, 0.6 s; Scale bar, 1 mm. (C) Outcome of the competition experiment. The bars represent standard deviations for at least four independently repeated experiments.

The laboratory *B. subtilis* strain 168 synthesizes only the enzymatically active GDH, RocG. The second GDH, GudB, which is encoded by the cryptic *gudB^CR^* gene, is enzymatically inactive. So far it has remained unclear why the inactive *gudB^CR^* gene is stably inherited in strain 168 in the lab over many passages. However, bacteria, which are equipped with reduced or elevated GDH activity might have a selective growth advantage when exogenous glutamate is scarce and present in excess, respectively. To address this question we performed an intraspecies competition experiment with strains BP40 (*rocG^+^ gudB^CR^*) and BP52 (*rocG^+^ gudB^+^*) (Figure 2A). Strain BP40 synthesizes only the active GDH RocG, while BP52 produces two active GDHs, RocG and GudB [11], [27]. To identify the survivors during and after co-cultivation of BP40 and BP52 by counting yellow and blue colonies, the strains were labelled with the fluorophore-encoding genes *yfp* and *cfp*, respectively (Figure 2A and Figure 2B).

Populations of the two strains that were mixed in a 1∶1 ratio were grown for a maximum of 24 h either in C-Glc minimal medium containing glucose and ammonium as source of carbon and nitrogen, respectively, or in CE-Glc medium containing glutamate as the additional nitrogen source. The competition experiment revealed that strain BP40 (*rocG^+^ gudB^CR^ yfp*), which is isogenic to the laboratory strain 168, outcompeted strain BP52 (*rocG^+^ gudB^+^ cfp*) expressing two functional GDHs, in the absence of exogenous glutamate (Figure 2C). Thus, a reduced amount of glutamate-degrading enzyme activity provides the bacteria with a selective growth advantage when the supply with external glutamate is low. Indeed, a *B. subtilis* strain expressing only *rocG* grew faster with a generation time of 58 min than a strain synthesizing two active GDHs (generation time of 83 min) in the absence of glutamate. It is safe to assume that high GDH activity is a drain for the intracellularly formed glutamate that could otherwise be used for anabolic purposes (see Figure 1A). By contrast, when external glutamate was available, strain BP52 (*rocG^+^ gudB^+^ cfp*) equipped with high amount of GDH activity outcompeted strain BP40 (*rocG^+^ gudB^CR^ yfp*), which expressed a single GDH-encoding gene. Under these growth conditions, high-level of GDH activity is obviously advantageous for the cell because two catabolically active GDHs, RocG and GudB, degrade glutamate faster than a single enzyme and the liberated 2-oxoglutarate may serve together with glucose as an extra source of energy. The fact that a *B. subtilis* strain synthesizing two active GDHs grew slower with exogenous glutamate (generation time of 60 min) than a strain synthesizing a single GDH (generation time of 53 min) is in line with this idea. Very similar observations were made when the experiments were repeated with reciprocally labelled strains BP41 (*rocG^+^ gudB^CR^ cfp*) and BP156 (*rocG^+^ gudB^+^ yfp*). Thus, neither the *cfp* gene nor the *yfp* gene influenced the outcome of the competition experiment. Moreover, we excluded that either of the two fluorophores CFP and YFP affected growth of the parent strain 168 (*rocG^+^ gudB^CR^*) or that of derivatives of strain GP804 (*rocG^+^ gudB^+^*), which has been used for the competition experiment (Figure S1). Thus, our results indicate that the amount of GDH activity strongly determines the fitness of the bacteria depending on the supply with external nitrogen. Moreover, the adaptation of *B. subtilis* for fast growth in glucose-ammonium minimal medium during its domestication seems to be indeed the reason for the inactivation of the *gudB^+^* allele (see below [25], [28]). However, the stable inheritance of the cryptic *gudB^CR^* allele in the laboratory strain 168 suggests that synthesizing at least one active GDH (RocG) is sufficient for optimal growth of the bacteria on complex medium.

### Lack of Exogenous Glutamate is the Driving Force for the Selection of Mutants that have Inactivated the *gudB* Gene

**Figure 3:**
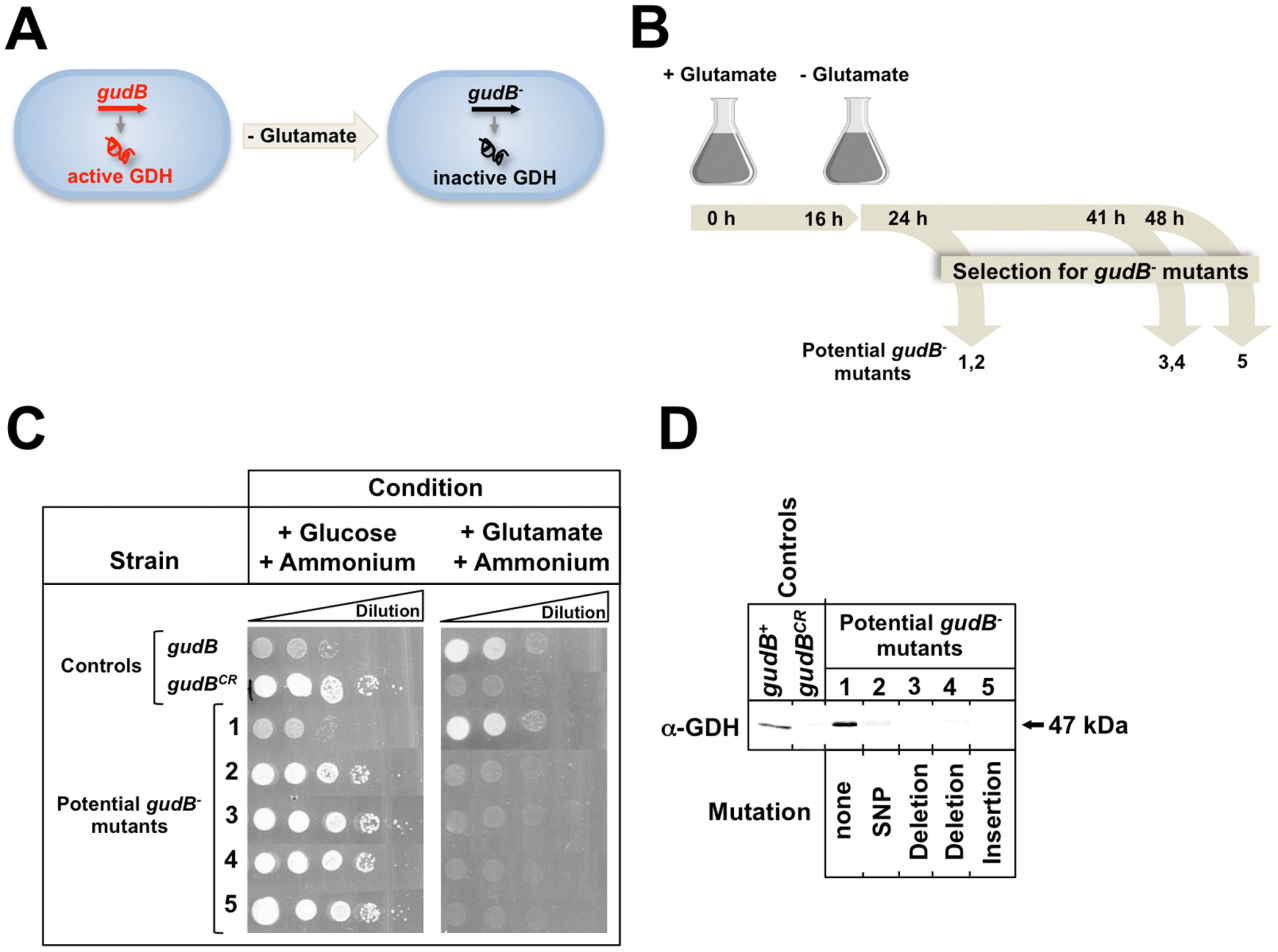
Isolation of *B. subtilis* mutants that have inactivated the *gudB* gene. (A) Lack of exogenous glutamate is the driving force allowing the selection of mutants with inactivated *gudB* alleles. (B) Prior to growth in the absence of glutamate, the *B. subtilis rocG^–^* mutant strain GP801 (*ΔrocG gudB^+^*) expressing only the active GDH, GudB was grown in C minimal medium supplemented with glucose and glutamate as carbon and nitrogen sources (plus glutamate), respectively. During growth in minimal medium lacking glutamate (no glutamate) samples were taken at indicated time points. (C) 5 µl were plated from serial dilutions (from non-diluted till 10^−6^) of cell suspensions of the *gudB^CR^* and *gudB^+^* control strains GP754 (*ΔrocG gudB^CR^*) and GP801 (*ΔrocG gudB^+^*), respectively, and the isolated *gudB^–^* mutants for phenotypic analyses. The dilutions were spotted on minimal medium agar plates supplemented either with glucose and ammonium or with glutamate and ammonium. The plates were incubated for 48 h at 37°C. (D) Western blot analysis to monitor synthesis of the GDH, GudB in the *gudB^–^* isolates using GDH-specific antibodies. Results of the sequence analysis of the *gudB^–^* alleles are summarized below (see Table 1).

As described above, “wild” wild-type isolates of *B. subtilis* express the two functional GDH-encoding genes *rocG* and *gudB*, while the *gudB* allele is cryptic in the laboratory strain 168 [11], [25]. It has been suggested that the *gudB* gene became cryptic during adaptation of *B. subtilis* for efficient growth with a poor nitrogen source such as ammonium (see above; [28]). Indeed, we have previously shown that only mutants of the laboratory strain 168, devoid of any glutamate-degrading GDH activity can grow in CS medium containing succinate and ammonium as poor sources of carbon and nitrogen, respectively, even though this strain possesses the genetic equipment for glutamate biosynthesis under these conditions [20]. Here we wanted to address the question whether poor carbon and nitrogen supply results in the selection of mutants, which have specifically inactivated the *gudB* gene encoding the active GDH, GudB (Figure 3A). For this purpose, we cultivated strain GP801 (*ΔrocG gudB^+^*) synthesizing only the active GDH GudB in C minimal medium supplemented with succinate and low amounts of glucose (0.05%), and the poor nitrogen source ammonium. During growth for a maximum of 48 h we took samples at three different time points as indicated in Figure 3B. The five samples that contained potential *gudB^–^* mutants, lacking GDH activity were propagated on CS agar plates. The strains GP801 (*ΔrocG gudB^+^*) and GP754 (*ΔrocG gudB^CR^*) served as negative control and positive controls, respectively. The CS plates were incubated for 48 h until single colonies appeared. As the cells in the five potential *gudB^–^* mutants grew as fast as the positive control it can be excluded that the inactivation of *gudB^+^* occurred on the CS plates (data not shown).

Next we isolated single colonies of the five potential *gudB^–^* isolates that were grown on the CS agar plates and evaluated growth of the isolates at conditions that require either the presence or the absence of the functional GDH, GudB (Figure 3B and Figure 3C). Strain GP754 (*ΔrocG gudB^CR^*) and the parent strain GP801 (*ΔrocG gudB^+^*) served as controls. The *gudB^–^*1 isolate, which was isolated early during cultivation, showed the phenotype of the GDH-proficient parent strain GP801 because it grew only poorly with glucose and ammonium as carbon and nitrogen sources, respectively. On the other hand, this isolate grew as well as the parent strain GP801 with glutamate as the single carbon and nitrogen source. It has been previously reported that *B. subtilis* is only capable of utilizing glutamate when *gudB* is encoding the enzymatically active GDH GudB [11]. Thus, isolate *gudB^–^*1 must still express the active *gudB^+^* gene. Indeed, sequencing and Western blot analyses revealed that the *gudB^+^* gene was intact and GudB was synthesized (Figure 3D and Table 1). It is very likely to assume that either the inactivated *gudB^–^*1 allele was mutated back to *gudB^+^*, encoding an active GDH or that the *gudB^–^*1 got lost during passaging of the isolates on rich medium. The *gudB^–^*2 isolate, which was isolated at the same time as the isolate *gudB^–^*1, as well as the isolates *gudB^–^*3, -4, and -5 from later time points grew like the GDH-deficient control strain GP754 (*ΔrocG gudB^CR^*) with glucose and ammonium as carbon and nitrogen sources, respectively (Figure 3C). Moreover, the fact that the isolates *gudB^–^*2, -3, -4, and -5 were not able to utilize glutamate as the single carbon source indicated that the *gudB^+^* gene was inactivated. Indeed, all four mutants had acquired different mutations, such as point mutations, deletions and insertions (see Table 1). As revealed by Western blot analysis, these mutations resulted in the absence of a functional GDH in the four *gudB^–^* mutants (Figure 3D). Thus, cultivation of *B. subtilis* in the absence of exogenous glutamate results in the emergence of mutants that have inactivated the single GDH-encoding gene to prevent degradation of glutamate.

**Table 1:** Mutations in the *gudB* gene of the *gudB^–^* isolates and biochemical consequences for the GudB mutant proteins.

Strain	Genotype	Time point ofisolation (h)	Mutation	AA exchange	Western blottingsignal
GP754	*gudB^CR^*	–	–	–	no
GP801	*gudB^+^*	–	Δ280-288	Δ94*VKA*96	yes
BP42	*gudB^–^1*	24	Δ280-288	Δ94*VKA*96	yes
BP44	*gudB^–^2*	24	Δ280-288, T896G	Δ94*VKA*96; *L*299*R*	no
BP48	*gudB^–^3*	41	Δ280-288, Δ766	Δ94*VKA*96; 256-277, 22 different amino acids;Δ278-419	no
BP46	*gudB^–^4*	41	Δ280-288, Δ673-738	Δ94*VKA*96, Δ225*VVQGFGNAG SYLAKFMHDAGAK*246	no
BP47	*gudB^–^5*	48	Δ280-288, insertion of C1222and T1223	Δ94*VKA*96; 406-437, 32 different amino acids	no

In the active *gudB^+^* allele the bases from 280 till 288 are deleted.

### Exogenous Nitrogen Strongly Affects Emergence and Clonal Expansion of the Active *gudB^+^* Allele in a Population of Cells, which Originates from a *ΔrocG gudB^CR^* Strain

The presence of the *gudB^+^* allele in a population of *ΔrocG* cells, encoding the enzymatically and regulatory active GDH GudB can be monitored indirectly using a translational *gltA-lacZ* fusion [13]. In cells lacking a functional GDH, the transcription factor GltC constitutively activates the transcription of the *gltA-lacZ* fusion. By contrast, during growth in SP rich medium GltC is unable to activate the *gltA* promoter in cells expressing either *rocG^+^* or *gudB^+^* because both active GDHs, either RocG or GudB can bind to and inactivate GltC [22]. Thus, colonies synthesizing the inactive GudB^CR^ enzyme or the active GDH GudB can be distinguished on SP agar plates supplemented with X-Gal to monitor the activity of a *gltA* promoter*-lacZ* fusion.

**Figure 4:**
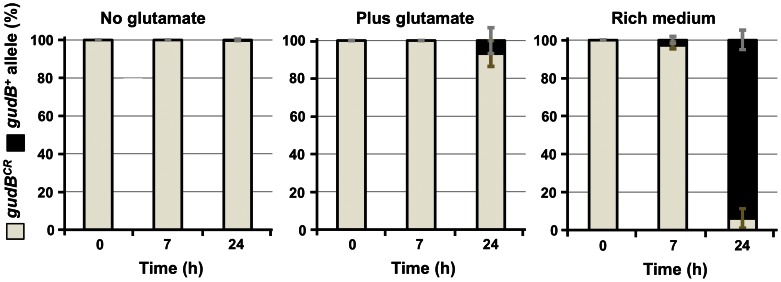
Effect of glutamate supply on the clonal expansion of *gudB* mutants. The *B. subtilis ΔrocG* mutant strain GP754 (*ΔrocG gudB^–^*) was grown in C minimal medium supplemented with glucose and ammonium (no glutamate), glucose and ammonium/glutamate (plus glutamate), and in SP (rich) medium. The bars represent standard deviations for four independently repeated experiments (see Table S5). The amount of *gudB^CR^* and *gudB^+^* mutants at the indicated time points are shown by light brown and black bars, respectively.

Using this approach we studied the emergence and clonal expansion of the *gudB^+^* allele in cells that were grown in complex and minimal medium with different quantities of nitrogen (Figure 4). For this purpose strain GP754 (*ΔrocG gudB^CR^ amyE::*(*gltA-lacZ*)) was grown over night in C-Glc medium at 37°C and used to inoculate either 10 ml SP rich medium or CE-Glc and C-Glc medium to an approximate OD_600_ of 0.1. C-Glc is minimal medium that contained glucose and ammonium as carbon and nitrogen sources, respectively. CE-Glc medium contained in addition to ammonium, glutamate as a nitrogen source. All cultures were grown for up to 24 h. Samples were taken and diluted to an appropriate cell titre, allowing to count single colonies (between 48 and 219 colonies per plate) on SP medium agar plates that were supplemented with X-Gal. Blue and white colonies that appeared after incubation of the plates over night express the cryptic *gudB^CR^* and the active *gudB^+^* allele, respectively (see Table S5 for raw data of the experiment). We observed that prior to growth under selective pressure (time point 0 h) all cells plated from each culture expressed the cryptic *gudB^CR^* allele (Figure 4). This was also true for all the cells that were cultivated for up to 24 h in C-Glc medium (no exogenous glutamate). Obviously, cells that express the cryptic *gudB^CR^* allele have a selective growth advantage over cells that express the active *gudB^+^* gene when no exogenous glutamate is provided to the cells. In the culture containing CE-Glc medium the active *gudB^+^* allele did not appear after 7 h of growth but about 4% of the cells in this culture expressed the active *gudB^+^* allele after 24 h of growth (Figure 4). Moreover, already 2% of the cells that were grown for 7 h in rich medium expressed the active *gudB^+^* allele and almost the complete cell population synthesized the active GDH, GudB after 24 h of cultivation. Thus, the suppressor mutants that have acquired the active *gudB^+^* gene by spontaneous mutation obviously had a strong selective growth advantage with excess glutamate that is present in CE-Glc and in rich medium, and the bacteria expressing this allele rapidly outcompeted those cells that had retained the *gudB^CR^* allele (Figure 4). The rapid propagation of *gudB^+^* mutants in the cell population is obviously driven by their capability of utilizing glutamate in addition to glucose as a carbon source. In contrast, the cells that express the cryptic *gudB^CR^* allele have a selective growth advantage over cells that expressed the mutated *gudB^+^* gene in the absence of glutamate (Figure 4). This suggests that the selective pressure acting on the *ΔrocG gudB^CR^* mutant strain GP754 lacking GDH activity is rather low when the supply with external nitrogen is low. Moreover, the few *gudB^+^* alleles that might have emerged by spontaneous mutation of the *gudB^CR^* allele in the population of cells obviously did not provide the bacteria with a selective advantage when exogenous glutamate was absent. Taken together, our observation suggests that external supply with glutamate strongly affects the clonal expansion of the *gudB^+^* gene in a population of cells but not its emergence.

### A GFP-based System to Monitor the State of the *gudB* Allele

**Figure 5:**
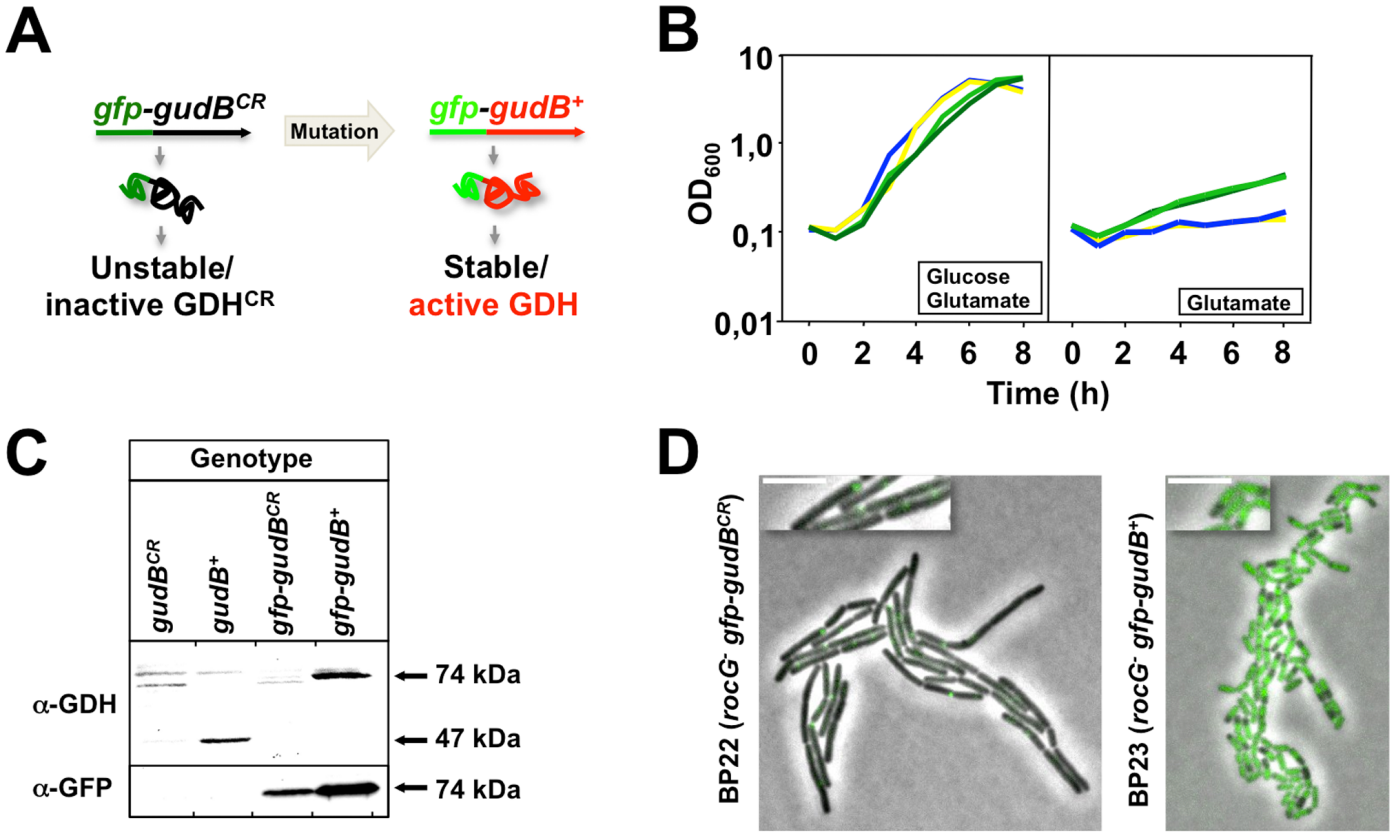
A GFP-based system to monitor the state of the *gudB* allele in *B. subtilis*. (A) The principle of the system is based on the stabilities of the inactive and active GudB^CR^ and GudB proteins, respectively. (B) Growth assay to confirm the enzymatic activity of the GFP-GudB fusion protein. C minimal medium supplemented with glucose and glutamate as the carbon and nitrogen sources, respectively, served as the positive control. Strains GP1165 (*ΔrocG gudB^+^*) and BP23 (*ΔrocG gfp-gudB^+^*) synthesizing the active GudB (dark green) and GFP-GudB (light green) alleles, respectively, were capable of catabolizing glutamate. The strains GP1163 (*ΔrocG gudB^CR^*) and BP22 (*ΔrocG gfp-gudB^CR^*) synthesizing the inactive *gudB^CR^* (yellow) and *gfp-gudB^CR^* (blue) alleles, respectively, did not grow with glutamate as the single source of carbon and nitrogen. (C) Western blot analysis to evaluate the stabilities of the inactive and active GudB^CR^ and GudB variants in strains GP754 (*ΔrocG gudB^CR^*) and GP801 (*ΔrocG gudB^+^*), respectively, using polyclonal antibodies raised against GDH and GFP. The corresponding GFP-GudB^CR^ and GFP-GudB fusion proteins are synthesized in strains BP22 (*ΔrocG gfp-gudB^CR^*) and BP23 (*ΔrocG gfp-gudB^+^*), respectively. (D) Fluorescence of microcolonies of strains BP22 and BP23 that express the cryptic *gfp-gudB^CR^* and the active *gfp-gudB^+^* fusion genes, respectively. Exposure time, 1 s; scale bar, 5 µm.

As mentioned above, the inactive GudB^CR^ protein is extremely unstable and subject to rapid proteolytic degradation [26]. In contrast to this, the enzymatically active GDH, GudB is stable [27]. These biochemical properties of the GudB variants stimulated us to develop a GFP-based system that would allow us to determine the state of the *gudB* allele in single cells and in an aging colony of *B. subtilis* (Figure 5A). For this purpose, we fused the *gfp* gene in frame either to the 5′ or the 3′ ends of the *gudB^CR^* and *gudB^+^* alleles. The 3′ and the 5′ gene fusions were integrated into the chromosome by a Campbell-type and double homologous recombination into the *amyE* gene, respectively. Previously, we have shown that the decryptification frequency of *gudB^CR^* was not affected when the gene was expressed from the *amyE* locus [27].

The *in vivo* activities of the different fusion proteins were examined by growth experiments (Figure 5B). The fusion of *gfp*, either to the 3′ or the 5′ end of the *gudB* variants did not affect growth of *B. subtilis* on complex and CE-Glc minimal medium containing glucose and glutamate as carbon and nitrogen sources, respectively. However, when glutamate was provided to the cells as the single carbon source, only strains GP1165 (*rocG* ^–^ *gudB^+^*) and BP23 (*rocG^–^ gfp-gudB^+^*) synthesizing GudB and GFP-GudB were capable of utilizing glutamate (Figure 5B). This observation is in perfect agreement with previous results showing that the capability of *B. subtilis* of growing with glutamate strictly depends on the presence of the active GDH, GudB (see Figure 3; [11]). Interestingly, fusion of the *gfp* gene to the 3′ end of the *gudB^+^* allele renders the encoded GudB-GFP fusion protein inactive (Figure S4). It has been shown that the C-termini of the six identical subunits are buried within the structures of the active GDHs, RocG and GudB [13]. Therefore it is likely that the 27 kDa GFP protein disrupts the integrity of the GudB structure and the activity of the enzyme.

As the system to monitor the state of the *gudB* allele in a population of cells or in single cells of *B. subtilis* is based on protein stability, we evaluated the stabilities of the GFP-GudB^CR^ and GFP-GudB variants in strains BP22 (*rocG^–^ gfp-gudB^CR^*) and BP23 (*rocG^–^ gfp-gudB^+^*), respectively, by Western blotting and fluorescence microscopy (Figure 5C and Figure 5D). The isogenic strains GP754 (*rocG^–^ gudB^CR^*) and GP801 (*rocG^–^ gudB^+^*) synthesizing the native GudB^CR^ and GudB variants, respectively, served as controls for the Western blotting experiment. Like the native GudB^CR^ variant, the GFP-tagged GudB^CR^ protein was unstable and cells showed a polar fluorescence signal (Figure 5C and Figure 5D). By contrast, the GFP-GudB fusion and the native GudB protein were stable and active, and cells expressing the *gfp-gudB^+^* allele were strongly fluorescent. Thus, the GFP-based system is a powerful tool to assess the state of the *gudB* allele in single cells of *B. subtilis*.

### Application of the GFP-based System to Monitor the State of the *gudB* Allele in Single Cells in an Aging Colony of *B. subtilis*

**Figure 6:**
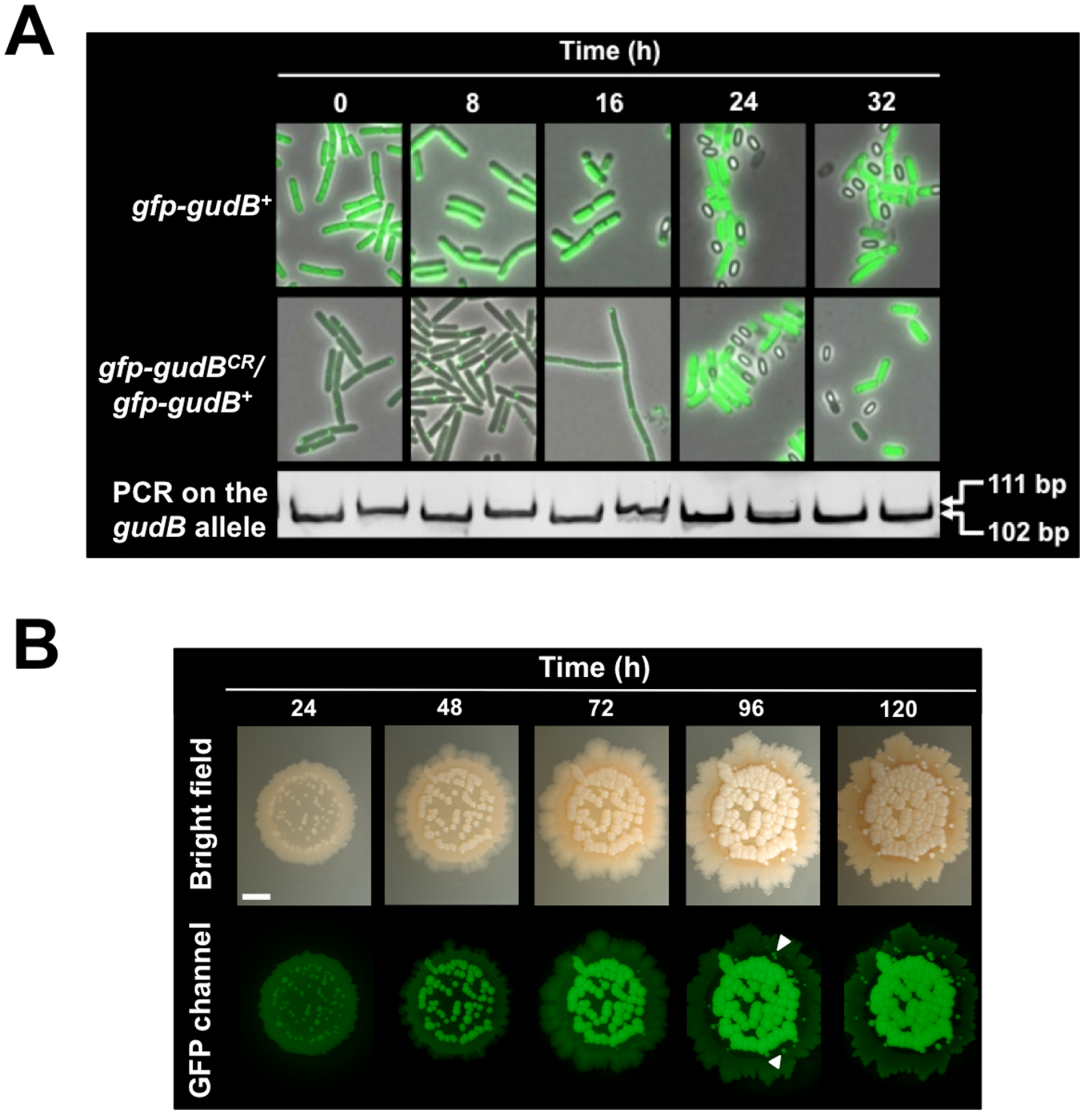
Direct visualization of the emergence and clonal expansion of the decryptified *gudB^+^* allele in *B. subtilis*. (A) Decryptification of the *gudB^CR^* allele and clonal expansion of the *gudB^+^* mutants over time in complex medium. Exposure time, 0.6 s; scale bar, 5 µm. The state of the DR in the *gudB^CR^* allele in strain BP22 (*ΔrocG gfp-gudB^CR^*) was analyzed by colony PCR and the DNA species were visualized by PAA gel electrophoresis (Figure S2). Strain BP23 (*ΔrocG gfp-gudB^+^*) expressing the active *gudB^+^* allele served as the positive control. (B) Emergence of *gfp-gudB^+^* suppressor mutants in a developing colony of strain BP22 (*ΔrocG gfp-gudB^CR^*) on rich medium. The white arrows indicate late suppressors. Exposure time, 2 s; scale bar, 2 mm.

We next used the GFP-based system to visualize the emergence and clonal expansion of the *gudB^+^* allele in single cells that were derived from *B. subtilis ΔrocG* mutant strain BP22 lacking the active GDH RocG but expressing the potentially mutatable cryptic *gfp-gudB^CR^* allele (see Figure 6A). For this purpose, we grew the strain over night in C minimal medium supplemented with glucose and ammonium. As shown above, under these growth conditions the proliferation of suppressor mutants expressing the decryptified *gudB^+^* gene is very low (see Figure 4). This culture was then used to inoculate SP rich medium to an approximate OD_600_ of 0.05. The isogenic strain BP23 (*ΔrocG gfp-gudB^+^*) expressing the active GFP-GudB fusion protein served as a positive control. The emergence of *gudB^+^* suppressor mutants was then followed over time for up to 32 h by fluorescence microscopy (Figure 6A). As a complementary approach to visualize the state of the *gudB* in cells obtained from the same culture, we performed colony PCR using a primer pair that hybridizes close to the DR repeat in the *gudB* gene. The 111 bp and 102 bp long DNA species derived from a *gudB^CR^* and a *gudB^+^* mutant, respectively, can easily be distinguished by polyacrylamide (PAA) gel electrophoresis (see Figure S2 and Materials and Methods). As shown in Figure 6A, single cells that were obtained from the control strain BP23 (*ΔrocG gfp-gudB^+^*) showed a strong fluorescence signal that was evenly distributed over the cells. This confirmed our previous observation that the active *gfp-gudB^+^* fusion gene that is missing one part of the 9 bp long DR is stably expressed in *B. subtilis* (see Figure 5C). By contrast, all cells of strain BP22 (*ΔrocG gfp-gudB^CR^*) expressing the cryptic *gfp-gudB^CR^* allele showed a polar fluorescence signal within the first 8 h of cultivation. Thus, the majority of cells expressed the *gudB^CR^* gene during this time. Indeed, the colony PCR on cells that were obtained from the same culture revealed that the complete population expressed the cryptic *gfp-gudB^CR^* allele because only the 111 bp but not the 102 bp long DNA species was visible by PAA gel electrophoresis (Figure 6A). After 16 h of incubation the cells expressing the mutatable *gfp-gudB^CR^* allele still showed a polar fluorescence signal. However, the colony PCR revealed that few cells expressed the decryptified *gudB* gene as the 102 bp long DNA species weakly appeared. This indicates that the mutagenesis of the cryptic *gudB^CR^* allele must have taken place during the first 16 h of cultivation and those cells expressing the active *gudB^+^* allele started to outcompete cells that did not harbour the active *gudB^+^* allele. By looking at samples after 24 h and 32 h of cultivation, we observed that all cells were strongly fluorescent. Thus, most of the cells that have emerged from strain BP22 (*ΔrocG gfp-gudB^CR^*) seemed to express the active *gfp-gudB^+^* allele. Indeed, the colony PCR revealed that the cryptic *gudB^CR^* allele completely disappeared from the cell population (Figure 6A).

Previously, it has been claimed that each suppressor mutant that has emerged from a *rocG^–^* mutant lacking GDH activity will have mutated the cryptic *gudB^CR^* [11], [27]. However, there are several examples showing that mutations that accumulate to suppress a strong phenotype might occur at different loci on the chromosome [30]. To verify that each suppressor of a *B. subtilis ΔrocG* mutant lacking the GDH RocG mutates the *gudB^CR^* gene and expresses the functional GDH GudB, we spotted cells of strain BP22 (*ΔrocG gfp-gudB^CR^*) on rich medium and followed the emergence and clonal expansion of suppressor mutants in an aging colony. As shown in Figure 6B, each emerging papilla, even papilla that appeared later, became fluorescent. Obviously, the selective pressure that is exerted on the *rocG* mutant lacking a GDH results in the rapid proliferation of suppressors that have specifically mutated the *gudB^CR^* gene as no phenotypically different suppressor emerged. Taken together, our fluorescence microscopic method, which is based on the stability of GFP-labelled GudB variants, is a powerful tool to monitor the emergence and clonal expansion of the *gudB^+^* allele in single cells and in an aging colony of *B. subtilis*.

### Other Perfect DRs Present on the *B. subtilis* Chromosome Remain Unaffected in Suppressor Mutants that have Mutated the Cryptic *gudB^CR^* Allele

**Figure 7:**
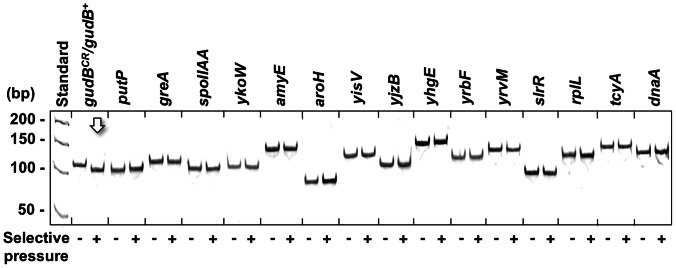
Stabilities of DRs during growth under strong selective pressure that is exerted on a *B. subtilis rocG^–^ gudB^CR^* mutant. The stabilities of DRs in a population of *gudB^+^* cells that originated from the *rocG^–^* mutant GP747 were analyzed by colony PCR.

Until now one factor, the Mfd protein that links transcription with DNA repair, was shown to be involved in the decryptification of the *gudB^CR^* gene [27]. However, the observation that the cryptic *gudB^CR^* gene is rapidly mutated with a high frequency of 10^−4^ in a *B. subtilis rocG^–^* mutant raised the question whether other loci on the *B. subtilis* chromosome that could be potentially mutated are modified by the same factor(s) during growth under strong selective pressure that is exerted on the *rocG^–^* mutant. To address this question, we studied the integrity of other DRs by colony PCR in a cell population that was derived from the *rocG^–^* mutant strain GP747 (*rocG^–^ gudB^CR^*). For this purpose the cells were cultivated in SP rich medium, conditions that result in the accumulation and clonal expansion of cells expressing the active *gudB^+^* allele in the population (see Materials and Methods). In addition to the DR of the *gudB^CR^* gene, 15 other DRs were identified using the tandem repeat database for bacteria (http://minisatellites.u-psud.fr/ASPSamp/base_ms/bact.php). The genes containing the DRs virtually cover the whole chromosome and the DRs are either in frame or not in frame but with the same total length 18 bp and a unit size of 9 bp as the DR in the *gudB^CR^* gene (Figure S3). Among the 15 genes are at least five that show expression profiles similar to that of the *gudB^CR^* gene (Table S4; [31], [32]). As shown in Figure 7, during growth under selection only those cells accumulated that harbour the active *gudB^+^* gene as none of the 15 other DRs was mutated in the population of *gudB^+^* cells. Thus, once the cryptic *gudB^CR^* allele is mutated the strong selective pressure that is exerted on the *rocG^–^* mutant leads to the rapid proliferation of cells synthesizing the active GDH, GudB (see Discussion). Moreover, even if the other DRs were mutated by spontaneous or adaptive mutagenesis in some *gudB^+^* cells of the population, these suppressor mutants synthesizing a functional GDH probably outcompeted those cells with mutations in other loci containing DRs due to their strong selective growth advantage.

### The Accumulation of a Mutated *gudB^+^* Allele Depends on the Selective Growth Advantage it Provides to a Cell

**Figure 8:**
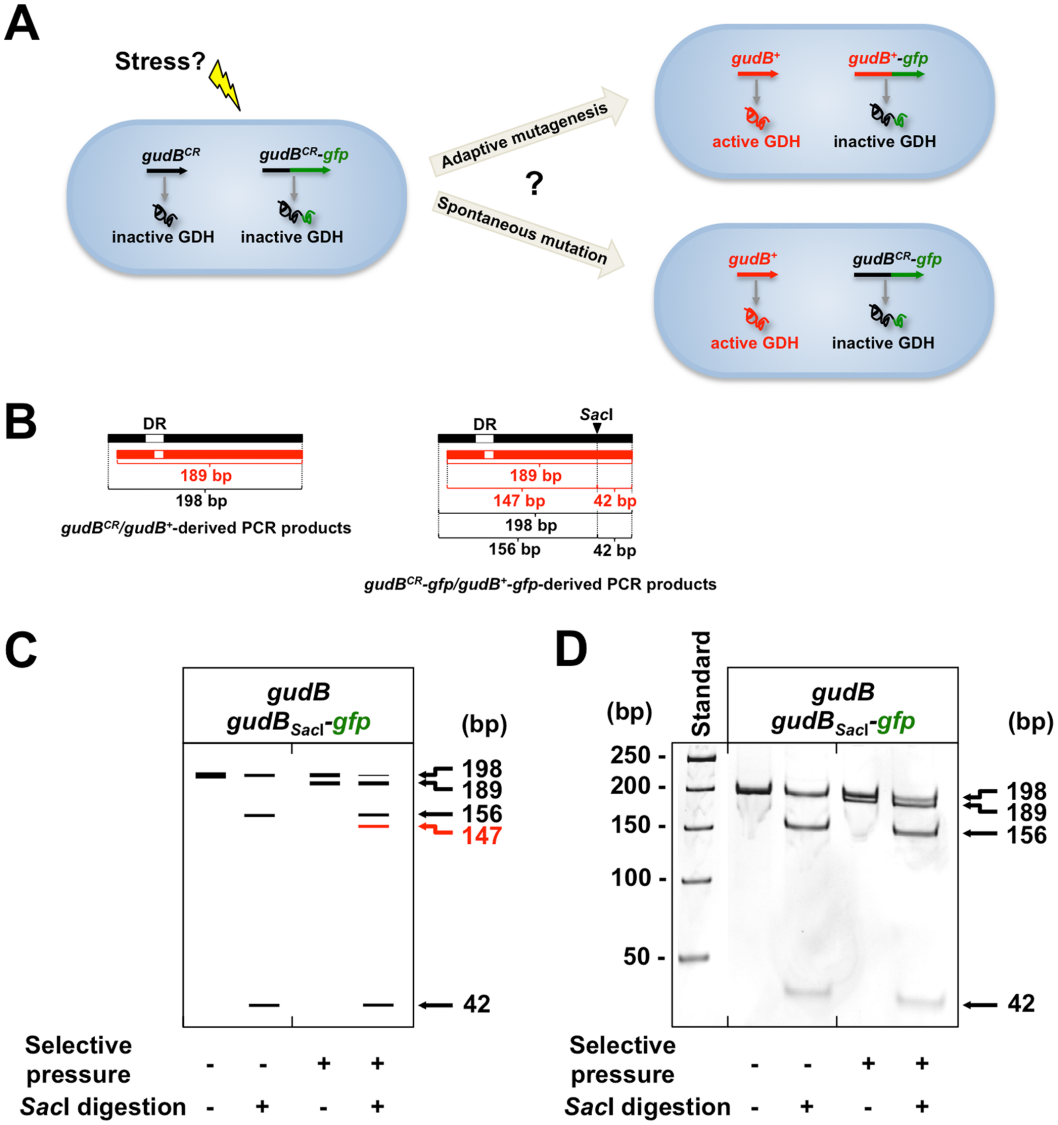
Stabilities of DRs present in the native *gudB^CR^* and in the *gudB^CR^_Sac_*_I_ *-gfp* alleles. (A) In addition to the native *gudB^CR^* allele, a second *gudB^CR^-gfp* fusion that could be potentially mutated during growth of a *B. subtilis ΔrocG* mutant under selective pressure was introduced into the *amyE* locus on the chromosome. (B) DNA species comprising the 9 bp DR were amplified by colony PCR using *gudB*-specific oligonucleotides (see Materials and Methods). To distinguish the DNA species derived from the two *gudB^CR^* alleles, a *Sac*I site was introduced into the *gudB^CR^-gfp* allele by exchanging G at position 402 by C. (C) Schematic illustration of the fragment pattern of DNA species obtained from cells collected prior to selective growth and after selection. The same samples were treated with *Sac*I. The emergence of a 147 bp DNA species shown by red letters would indicate the decryptification of the *gudB^CR^-gfp* allele. (D) Fragment pattern of DNA species obtained from real samples.

We have observed that in the background of a *rocG^–^* mutant strain, the native *gudB^CR^* gene, the *gfp-gudB^CR^* fusion gene but not the *gudB^CR^*-*gfp* fusion gives rise to an active GDH and provides the cell with a selective advantage (Figure S4). To address the question whether the strong selective pressure that is exerted on the *rocG^–^* mutant results in the adaptive mutagenesis or even directed mutagenesis of the DR present in the *gudB^CR^* gene, we thought to analyze the stability of the DRs in the native *gudB^CR^* allele and the *gudB^CR^-gfp* allele in cells, originating from a *rocG^–^* mutant (Figure 8A). For this purpose we introduced the *gudB^CR^-gfp* allele together with the *gudB* promoter into the *amyE* locus of the *rocG^–^* mutant strain GP747 giving strain BP31. Previously, we have shown that the decryptification of the *gudB^CR^* gene does not depend on its position on the chromosome. The modification of the 9 bp-long DRs in the two alleles during growth under strong selective pressure (growth in SP rich medium) that is exerted on the *rocG^–^* mutant was analyzed by colony PCR (see Materials and Methods). The DNA molecules derived from the *gudB^CR^* and *gudB^CR^-gfp* alleles were made distinguishable by introducing a *Sac*I restriction site into the *gudB^CR^-gfp* allele without changing the amino acid sequence in the translated protein (Figure 8B and Figure S5). Digestion of PCR products derived from this allele would shorten them by 42 bp (see schemes in Figure 8B and Figure 8C). Strain BP31 (*ΔrocG gudB^CR^ amyE::*(*gudB^CR^_Sac_*_I_ *-gfp*)) was grown in rich medium to select for cells expressing the *gudB^+^* alleles and PCR products were generated from cell samples that were collected prior to growth and after growth under selective pressure. The DNA molecules were analyzed by PAA gel electrophoresis. A single DNA fragment was observed in the PCR sample generated from cells prior to growth under selective pressure (Figure 8D). Two bands occurred upon *Sac*I digestion, indicating that DNA molecules derived from the native *gudB^CR^* allele and the *gudB^CR^_Sac_*_I_ *-gfp* allele were present in the mixture. In the PCR mixture that was generated from cells collected after growth under selective pressure, we identified two DNA species that might have been derived from cryptic and decryptified *gudB^+^* and *gudB^+^_Sac_*_I_ *-gfp* alleles (Figure 8D). After *Sac*I digestion only four instead of five DNA species occurred. If both alleles were mutated, we would have expected to observe 198 bp and 189 bp DNA species derived from the native allele, and 156 bp, 147 bp and 42 bp DNA species derived from the *gudB^CR^_Sac_*_I_ *-gfp* allele (Figure 8D). However, the 147 bp DNA molecule that could have originated from the decryptified *gudB^+^_Sac_*_I_ *-gfp* allele was missing (Figure 8C and Figure 8D). We excluded that the *Sac*I recognition site interferes with the activation of the *gudB^CR^_Sac_*_I_ allele (data not shown). Thus, only the native allele whose mutated form provides the bacteria with a strong selective growth advantage spread in the culture, while the mutated *gudB^CR^_Sac_*_I_ *-gfp* allele did not (Figure 8D).

## Discussion

Soil bacteria, such as *B. subtilis* live in a constantly changing environment. In principle, there are two different possibilities of how a living cell can respond and adapt to an environmental stimulus, *i.e*., a change in nutrient supply. On one hand bacteria can adjust their metabolism either by differential regulation of gene expression or by controlling the flux through central metabolic pathways [32], [33], [34]. On the other hand, mutational events may cause the accumulation of beneficial mutations and provide the bacteria with a selective growth advantage under a specific environmental condition [35]–[37]. Although the accumulation of mutations can also be detrimental for the bacteria, it is often the last option to ensure survival or growth in a specific environment if the regulatory infrastructure of the cell is exhausted. Indeed, recently we found that in a *rocG* ^–^ mutant strain, lacking GDH activity, the cryptic *gudB^CR^* gene is rapidly mutated with a high frequency of 10^−4^, and the suppressor mutants synthesize the enzymatically and regulatory active GDH, GudB [11], [27]. Although mutation frequencies in the range of 10^−4^ and even higher have been described in many other bacteria it is the highest frequency that has been described so far for *B. subtilis* [27], [38]. One attractive explanation for the high mutation frequency of the *gudB^CR^* allele might be that the lack of GDH activity due to the inactivation of the *rocG* ^+^ gene causes the stress-induced mutation of the *gudB^CR^* gene. The lack of GDH activity has several implications for the bacteria. First, in the absence of a functional GDH, the transcription factor GltC is highly active and constitutively activates transcription of the GOGAT-encoding genes [20], [22]. Second, a *rocG* ^–^ mutant cannot fully metabolize nitrogen sources such as arginine and ornithine that end up at the level of glutamate [11]. Moreover, either the accumulation of glutamate or of intermediates of the arginine-degradation pathway may be toxic for the cell. This seems to be indeed the case, as we have observed that *B. subtilis* is unable to grow with arginine in the absence of a functional GDH (data not shown). Finally, a very recent study has revealed that a *B. subtilis* mutant lacking GDH activity is more sensitive to β-lactam antibiotics [39]. Altogether, it seems to be an attractive idea that the pleiotropic phenotype of a *rocG* ^–^ mutant might cause the stress-induced activation of the *gudB^CR^* gene.

An observation 25 years ago suggested that bacteria respond to “stress” by directly modifying particular genes, and thereby speed up their own evolution [35]. This idea has been faced with scepticism as it implies the existence of a stress-sensing machinery, which transduces the selective pressure that is exerted on a maladapted organism to a specific locus on the chromosome [35], [40]. However, the existence of a molecular machinery that can anticipate which genomic alteration would provide the cell with a selective growth advantage is hard to imagine. Indeed, the genetic system developed by Cairns that suggested genomic adaptation by directed mutagenesis can be fully explained by spontaneous mutation and growth under selection [41]. However, non-spontaneous but stress-induced adaptive mutagenesis is exceedingly well-documented in both bacterial and human cells and many factors that are involved in stress-induced adaptive mutagenesis have been identified in the meantime [42]–[45]. Recently, a large network comprising more than 90 genes was shown to be involved in stress-induced mutagenesis as a result of DNA double-strand breaks in *E. coli* [46]. This observation illustrates the complexity of how environmental or endogenous “stress” exerted on maladapted cells may stimulate factors, which in turn enable the cells to accelerate their own evolution. However, recent microarray analyses did not reveal that any DNA-modifying factors, which might be required for the decryptification of the *gudB^CR^* gene, are induced by “stress” due to the lack of GDH activity in a *B. subtilis rocG^–^* mutant strain [27], [39], [47].

A plausible explanation for the rapid emergence and clonal expansion of the active *gudB* ^+^ allele in a population of cells could be that, if the allele once occurred by spontaneous mutation, those cells that harbour the *gudB* ^+^ allele have a strong selective growth advantage over the parent strain. Indeed, *rocG* ^–^ mutants expressing the *gudB* ^+^ allele showed a selective growth advantage in a strictly glutamate-dependent manner. The presence of exogenous glutamate strongly enhances growth of *gudB* ^+^ suppressors (see Figure 4). Obviously, cells synthesizing the enzymatically active GDH GudB can use glutamate as an additional source of energy, which drives the rapid clonal expansion of the suppressors. By contrast, a *B. subtilis* mutant devoid of GDH activity has a growth advantage when exogenous glutamate is not available. Under these conditions the endogenously synthesized glutamate can be used for anabolism instead of being degraded by a GDH and fed into carbon metabolism (Figure 1A). These interpretations are in perfect agreement with the results of our growth experiments and the competition experiments, showing that the fitness of *B. subtilis* equipped with different levels of GDH activity is determined by the availability of glutamate (see Figure 2C). Thus, the rapid emergence of the active *gudB* ^+^ allele can be explained by spontaneous mutation and growth under selection. However, the 9 bp-long DR that is present in the cryptic *gudB^CR^* gene seems to be a crucial element for the high mutation frequency of the gene. Indeed, mutations affecting the integrity of the DR in the *gudB^CR^* gene without changing the coding sequence resulted in a 15-fold reduced mutation frequency [27]. It is well-documented that DRs present in the genomes of both pro- and eukaryotes are hypermutable loci [38], [48], [49]. Thus, the DR in the *gudB^CR^* allele is a mutational hot spot that is essential for the rapid decryptification of the *gudB^CR^* allele in the background of a *rocG* ^–^ mutant.

There are several other prominent genetic systems that seemed to show stress-induced mutagenesis [41]. One example is the Rif^R^ system describing the accumulation of rifampicin-resistant (Rif^R^) mutants in aging, non-growing colonies of enteric bacteria [50]–[52]. Rif^R^ mutants have a selective growth advantage due to the accumulation of mutations in the *rpoB* gene, encoding the β-subunit of the RNA polymerase. This phenomenon has been first explained by stress-induced mutagenesis. However, as we have observed for the *gudB* system in this study, the accumulation of the Rif^R^ mutants is due to growth under selection and not stress-induced mutagenesis [50]. There are two similarities between our system and the Rif^R^ system. First, both the Rif^R^ and *gudB^+^* mutants appear with an uneven spatial distribution in the aging colony (see Figure 6). This observation suggests that the emergence of the beneficial mutations is rather spontaneous and driven by selection. In contrast, a globally enhanced mutation rate would result in the accumulation suppressor mutants that are evenly distributed over the aging colony. Second, neither the Rif^R^ nor the *gudB^+^* mutants show an increased frequency of second site mutations (Figure 7 and Figure 8). Taken together, our system and many other prominent genetic systems that appeared to support the idea of stress-induced adaptive mutagenesis can in fact be explained by growth under selection [41], [50]. The present study also revealed that the strength of the selective pressure that is exerted on a maladapted bacterium strongly affects the apparent mutation frequency of a mutational hot spot.

Our current research focus is aimed at the understanding of the molecular mechanism of the decryptification of the *gudB^CR^* gene and the role of the transcription-coupling repair factor Mfd, the only protein that has been identified to be involved in this process [27]. Moreover, it will be interesting to study the role of transcription in the mutation of the *gudB^CR^* gene. Finally, using the GFP-based system, which is based on the stability of the GudB variants, we aim to identify the proteolytic machinery that is involved in the rapid degradation of the inactive GDH, GudB, which is the most-unstable protein in *B. subtilis* [26].

## Materials and Methods

### Construction of Plasmids and Bacterial Strains

The plasmids (Table S2) of this study were constructed using oligonucleotides that are listed in Table S1. Plasmid DNA was extracted using the Nucleospin Extraction Kit (Machery and Nagel, Germany). Commercially available restriction enzymes, T4 DNA ligase and DNA polymerases were used as recommended by the manufacturers. PCR products and DNA fragments isolated from agarose gels were purified using the PCR purification Kit (Qiagen, Hilden, Germany). DNA sequences were determined by the dideoxy chain termination method (SeqLab, Göttingen, Germany). The plasmid pAC5 was used to express *gudB* alleles from the *amyE* locus in *B. subtilis* [53]. Plasmids pCFPbglS and pYFPbglS served as templates for PCR to amplify the fluorophore-encoding *cfp* and *yfp* genes, respectively [54]. The plasmid pGP1870 was used for the construction of a *gudB-gfp* fusion [55] (Table S2).

The *B. subtilis* strains used in this study are derivatives of strain 168 *trp^–^*. All strains were constructed by transformation according to the two-step protocol [56] using chromosomal or plasmid DNA (Tables S2 and S3). Transformants were selected on SP plates supplemented with the appropriate antibiotics. Chromosomal DNA was isolated as described previously [56].

Correct integration of DNA constructs into the *amyE* locus of the *B. subtilis* chromosome was verified by monitoring amylase activity. The activity of this enzyme was detected after growth on plates containing nutrient broth (7.5 g/l), 17 g Bacto agar/l (Difco) and 5 g hydrolyzed starch/l (Connaught). Starch degradation was detected by sublimating iodine onto the plates.

### Growth Conditions

*E. coli* or *B. subtilis* were grown in LB and SP medium or in C minimal medium supplemented with carbon sources, nitrogen sources and auxotrophic requirements (at 50 mg/l) as indicated [22], [56], [57]. CSE medium is C minimal medium supplemented with 0.6% (w/v) succinate and 0.8% (w/v) glutamate together with ammonium as basic sources of carbon and nitrogen, respectively [18]. C-Glc medium is C minimal medium supplemented with 0.5% (w/v) glucose. LB and SP plates were prepared by the addition of 17 g Bacto agar/l (Difco) to LB and SP (8 g nutrient broth/l, 1 mM MgSO_4_, 13 mM KCl, supplemented after sterilization with 2.5 µM ammonium ferric citrate, 500 µM CaCl_2_, and 10 µM MnCl_2_), respectively. When required, media were supplemented with antibiotics at the following concentrations: kanamycin (10 µg/ml), chloramphenicol (5 µg/ml) and spectinomycin (150 µg/ml).

### Competition Experiments

For the competition experiment, the bacteria were grown over night in LB medium at 28°C, diluted to an OD_600_ of 0.05 in either C-Glc or CE-Glc minimal medium, and mixed 1∶1 with the competitor strain in 20 ml of media in a 100 ml flask. The cultures were incubated at 37°C with agitation. The cells obtained by sampling at defined time points were diluted in a 0.9% saline solution up to 10^−3^ and 100 µl of the dilutions were plated on SP medium agar plates. The plates were incubated over night at 37°C and the surviving cells were visualised by stereo fluorescence microscopy. Each competition experiment was repeated at least four times. Transcription of the fluorophore genes is driven by the constitutively active *gudB* promoter [27] (Table S2).

### Isolation of *gudB^–^* Mutants

Strain GP801 (*ΔrocG gudB^+^*) synthesizing a single active GDH, GudB was first grown over night in LB medium at 30°C. Next day this culture was used to inoculate CSE-Glc minimal medium supplemented with succinate/glucose and ammonium/glutamate as carbon and nitrogen sources, respectively, to an approximate OD_600_ of 0.1. After propagation of strain GP801 for 16 h at 37°C the cells were diluted a second time to an OD_600_ of 0.05 in CS-Glc medium containing 10-fold less glucose, and ammonium as the single nitrogen source. After growth for 8 h a sample was taken and the cells were propagated on C-Glc medium supplemented with 0.5% glucose. The remaining cells were again diluted in CS-Glc medium containing 0.05% glucose and ammonium as carbon and nitrogen sources, respectively, and further incubated for up to 48 h. Samples taken after 41 h and 48 h of incubation were treated as the first sample.

### Western Blotting

For Western blot analyses proteins present in 15 µg cell free crude extract were separated by 12% SDS PAGE and transferred onto polyvinylidene difluoride membranes (BioRad) by electroblotting. RocG and GFP polyclonal antibodies were diluted 1∶15000 and 1∶10000, respectively and served as primary antibodies ([22]. MBL, Medical & Biological Laboratories). The antibodies were visualized by using anti-rabbit immunoglobulin alkaline phosphatase secondary antibodies (Promega) and the CDP-Star detection system (Roche Diagnostics), as described previously [22].

### Analysis of Direct Repeat Integrity

Deletions of single repeat units of 9 bp-long perfect and imperfect direct repeats that are present on the *B. subtilis* chromosome (Figure S3 & Table S4) were detected by colony PCR. Briefly, we designed oligonucleotides that hybridize 20–120 bp upstream and 20–120 bp downstream of the tandem repeats (Table S1). The oligonucleotides were used to generate 80–140 b long DNA fragments by colony PCR. The deletion of a single repeat unit in a gene containing a 9 bp-long tandem repeat would give rise to a 9 bp smaller PCR product as illustrated for the *gudB* locus (Figure S2). To monitor the integrity of direct repeats, we grew the bacteria over night in LB medium. Next day, an aliquot of the preculture was collected for colony PCR and the remaining cells were used to inoculate SP liquid medium to an OD_600_ of about 0.1. After 42 h of incubation, we collected another aliquot for colony PCR. Template DNA for PCR was generated by heating 100 µl of a *B. subtilis* cell suspension (usually about 1.5×10^8^ CFU/ml) for 10 min at 98°C. The cell titre was determined by counting the colony forming units obtained from serial dilutions that were propagated on SP medium agar plates. 2.5 µl of the heated cells served as template DNA in a 50 µl PCR. PCR products were separated by 15% polyacrylamide (PAA) gel electrophoresis using TAE running buffer and DNA molecules were visualized by ethidium bromide staining. Template DNAs that were isolated from cell populations harbouring either the inactive *gudB^CR^* or the active *gudB^+^* allele, or both alleles gave rise to 111 bp and 102 bp DNA species (Figure S2).

### Fluorescence Microscopy

For fluorescence microscopy, cells were grown in LB medium to optical densities as indicated, harvested, and resuspended in phosphate-buffered saline (pH 7.5; 50 mM). Fluorescence images were obtained with an Axioskop 40 FL fluorescence microscope, equipped with digital camera AxioCam MRm and AxioVision Rel (version 4.8) software for image processing (Carl Zeiss, Göttingen, Germany) and Neofluar series objective at ×100 primary magnification. The applied filter set was eGFP HC-Filterset (band-pass [BP] 472/30, FT 495, and long-pass [LP] 520/35; AHF Analysentechnik, Tübingen, Germany) for GFP detection. All images were taken at the same exposure times. The overlays of fluorescent and phase-contrast images were prepared for presentation with Adobe Photoshop Elements, version 8.0 (Adobe Systems, San Jose, CA). Pictures of developing *B. subtilis* colonies or aged colonies were taken with a stereo fluorescence microscope Lumar.V12 (Zeiss, Jana) equipped with the ZEN lite 2011 (blue edition) software. The applied filter sets were Lumar 46, 47 and 38 for YFP, CFP and GFP detection, respectively (Zeiss, Jena). Images were taken for up to 120 h at room temperature.

### Growth of Microcolonies on Agarose Slides

To prepare single cells of *B. subtilis* for outgrowth into microcolonies, LB precultures were grown over night at 30°C. Next day, the precultures were used to inoculate 10 ml SP liquid medium at an OD_600_ of about 0.05. At mid-exponential growth phase the cultures were diluted to an OD_600_ of 0.035 using SP liquid medium that has been 30-fold diluted with C minimal medium and the cells were spotted onto microscope slides for fluorescence microscopy [22], [58].

### Monitoring the Emergence of the *gudB* Allele by a Blue-white Screening System

The emergence of the *gudB* allele encoding the enzymatically and regulatory active GDH GudB can be monitored indirectly using a translational *gltA-lacZ* fusion [13]. In cells lacking a functional GDH, the transcription factor GltC constitutively activates the transcription of the *gltA-lacZ* fusion. By contrast, in cells expressing either *rocG* or *gudB* GltC is unable to activate the *gltA* promoter because both active GDHs, either RocG or GudB, can bind to and inactivate GltC [22]. Thus, colonies synthesising the inactive GudB^CR^ enzyme or the active GDH GudB can be distinguished on SP agar plates supplemented with X-Gal to monitor the *gltA-lacZ* fusion. The amount of *gudB^CR^* and *gudB* clones in a growing culture was determined by plating a countable number of cells on SP-X-Gal plates.

## Supporting Information

Figure S1 Effect of the *yfp* and *cfp* fluorophore genes on growth of *B. subtilis*. Mixed populations of strains BP40 (*rocG^+^ gudB^CR^ amyE::yfp*) and BP41 (*rocG^+^ gudB^CR^ amyE::cfp*) or BP52 (*rocG^+^ gudB^+^ amyE::cfp*) and BP156 (*rocG^+^ gudB^+^ amyE::yfp*) were grown for up to 24 h in C minimal medium supplemented with glucose and ammonium (C-Glc), and C-Glc minimal medium supplemented with glutamate. Prior to co-cultivation (0 h), and after 7 h and 24 h of growth dilutions of cells were plated on complex medium. The surviving cells that emerged after 12 h of incubation were identified by fluorescence microscopy and counted. The bars represent standard deviations for at least four independently repeated experiments.
(TIF)

Figure S2 Analysis of DR integrity in cell population. (A) Schematic illustration of the colony PCR to detect deletion of the 9 bp-long single repeat unit of the *gudB^CR^* DR (see Materials and Methods). KG166 and KG167 are forward and reverse oligonucleotides, respectively, that hybridise close to the tandem repeat of the *gudB^CR^* gene. (B) Evaluation of the method to analyse the state of *gudB* in a population of cells. The DNA molecules were generated by colony PCR using template DNA from *B. subtilis* strains GP342 (*gudB^CR^*) and GP801 (*gudB^+^*). The 1∶1 mixture of co-cultivated strains GP342 and GP801 was analysed by colony PCR to detect the presence of the *gudB^CR^* and *gudB* alleles in a population of cells. The 50 bp Gene Ruler (Thermo scientific, #SM0373) served as DNA ladder.
(TIF)

Figure S3 Location and direction of 16 genes with 9 bp-long DRs on the *B. subtilis* chromosome. Genes that are highlighted in red and green are encoded on the plus and minus strand, respectively. The circular map of the *B. subtilis* chromosome was generated using the open source BLAST Ring Image Generator software 0.95 (http://sourceforge.net/projects/brig/). The genes were positioned according to the Subtilist database (http://genolist.pasteur.fr/SubtiList/).
(TIF)

Figure S4 *In vivo* activities of GudB^CR^ and GudB variants fused to GFP. 5 µl were plated from serial dilutions (from 10^−1^ till 10^−6^) of cell suspensions of the control strains GP1163 (*rocG^+^ gudB^CR^*) and GP1165 (*rocG^+^ gudB^+^*), and the strains BP22, BP23, BP9 and BP10 expressing the *gfp-gudB^CR^, gfp-gudB^+^, gudB^CR^-gfp* and *gudB^+^-gfp* fusions, respectively. The dilutions were spotted on SP medium agar plates (rich medium), and C minimal medium supplemented either with glucose and glutamate (CE-Glc medium) or with glutamate and ammonium (CE medium). The plates were incubated for 48 h at 37°C.
(TIF)

Figure S5 Part of the sequence of the *gudB^CR^_Sac_*_I_ *-gfp* allele. The recognition site GAGCTC for the restriction endonuclease *Sac*I that is highlighted in green was generated by modification of the CTG leucine codon at position 402 to the leucine codon CTC (see Table S2). The 9 bp DR of the *gudB^CR^* gene is highlighted in red. Letters highlighted in black and pink indicate the regions where the oligonucleotides KG166 and KG196 hybridise.
(TIF)

Table S1 Oligonucleotides.
(DOCX)

Table S2 Plasmids.
(DOCX)

Table S3 *B. subtilis* strains.
(DOCX)

Table S4 9 bp long tandem repeats present in essential (indicated by a superscript “e”) and non-essential genes of the *B. subtilis* chromosome.
(DOCX)

Table S5 Raw data of the experiment shown in Figure 4.
(DOCX)
