# Supplementary figures and images for: Selection-Driven Accumulation of Suppressor Mutants in Bacillus subtilis: The Apparent High Mutation Frequency of the Cryptic gudB Gene and the Rapid Clonal Expansion of gudB+ Suppressors Are Due to Growth under Selection
Source: PLoS One. 2013 Jun 13;8(6):e66120. doi: 10.1371/journal.pone.0066120 (PMC3681913; doi:10.1371/journal.pone.0066120)

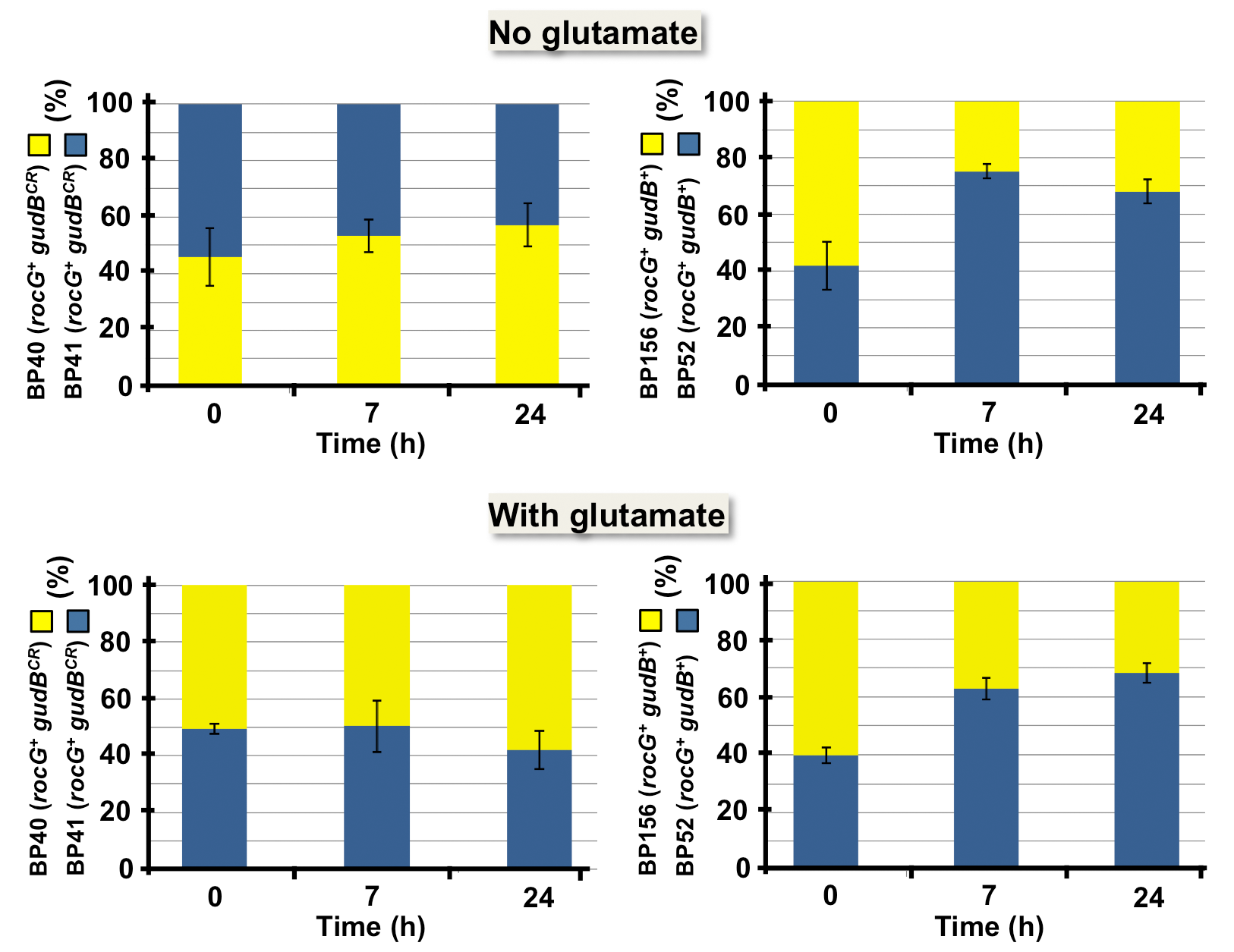

Supplement: Figure S1 — Effect of the yfp and cfp fluorophore genes on growth of B. subtilis. Mixed populations of strains BP40 (rocG+ gudBCR amyE::yfp) and BP41 (rocG+ gudBCR amyE::cfp) or BP52 (rocG+ gudB+ amyE::cfp) and BP156 (rocG+ gudB+ amyE::yfp) were grown for up to 24 h in C minimal medium supplemented with glucose and ammonium (C-Glc), and C-Glc minimal medium supplemented with glutamate. Prior to co-cultivation (0 h), and after 7 h and 24 h of growth dilutions of cells were plated on complex medium. The surviving cells that emerged after 12 h of incubation were identified by fluorescence microscopy and counted. The bars represent standard deviations for at least four independently repeated experiments. (TIF) [file pone.0066120.s001.tif]

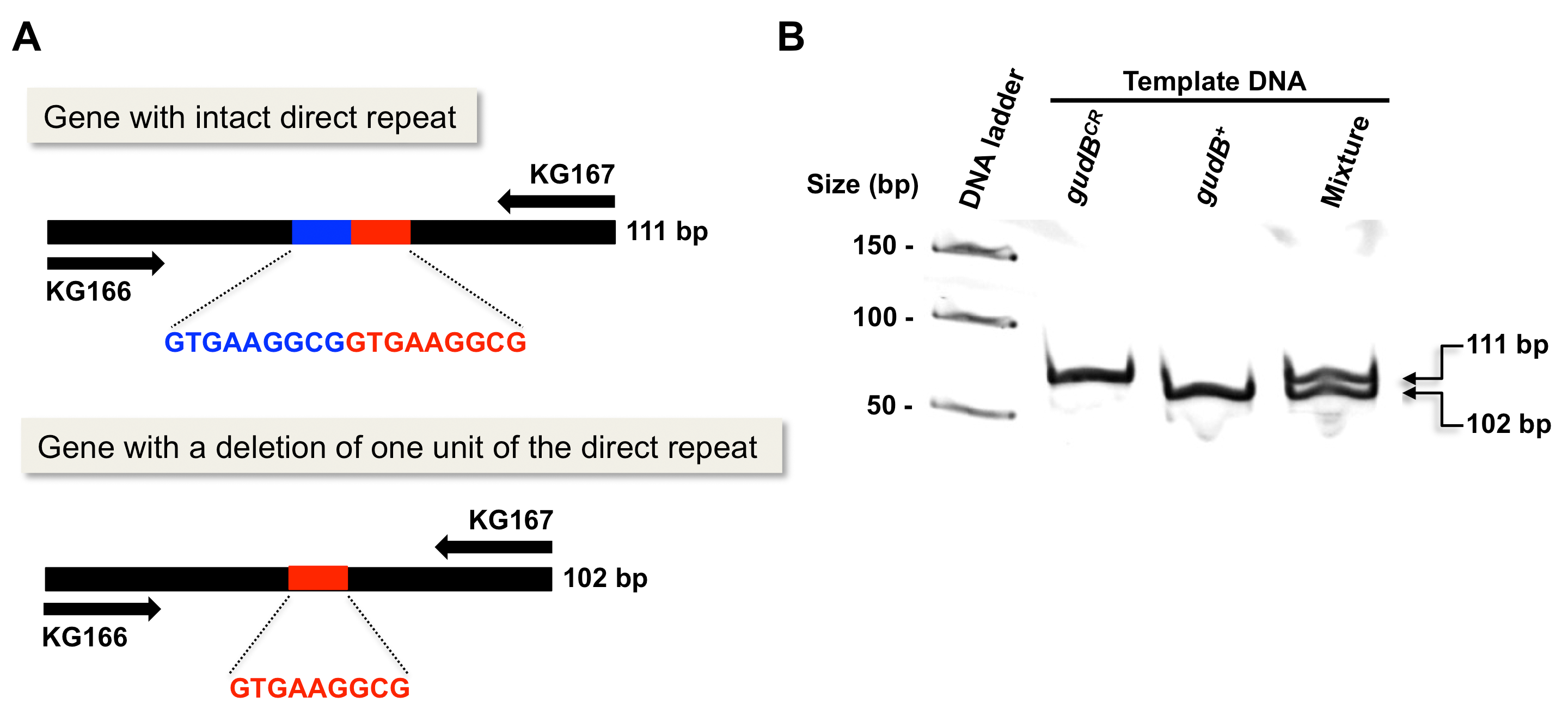

Supplement: Figure S2 — Analysis of DR integrity in cell population. (A) Schematic illustration of the colony PCR to detect deletion of the 9 bp-long single repeat unit of the gudBCR DR (see Materials and Methods). KG166 and KG167 are forward and reverse oligonucleotides, respectively, that hybridise close to the tandem repeat of the gudBCR gene. (B) Evaluation of the method to analyse the state of gudB in a population of cells. The DNA molecules were generated by colony PCR using template DNA from B. subtilis strains GP342 (gudBCR) and GP801 (gudB+). The 1∶1 mixture of co-cultivated strains GP342 and GP801 was analysed by colony PCR to detect the presence of the gudBCR and gudB alleles in a population of cells. The 50 bp Gene Ruler (Thermo scientific, #SM0373) served as DNA ladder. (TIF) [file pone.0066120.s002.tif]

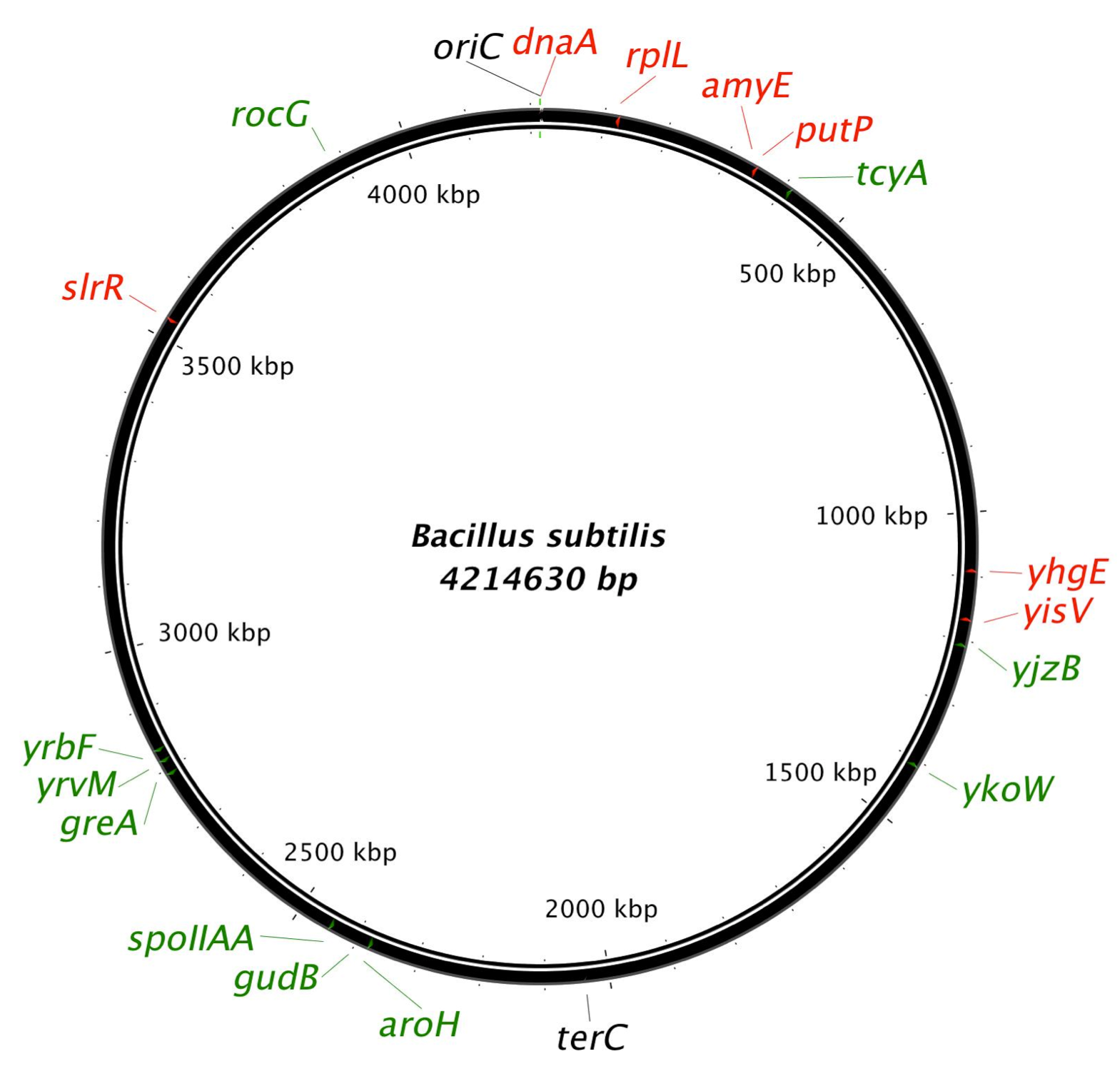

Supplement: Figure S3 — Location and direction of 16 genes with 9 bp-long DRs on the B. subtilis chromosome. Genes that are highlighted in red and green are encoded on the plus and minus strand, respectively. The circular map of the B. subtilis chromosome was generated using the open source BLAST Ring Image Generator software 0.95 (http://sourceforge.net/projects/brig/). The genes were positioned according to the Subtilist database (http://genolist.pasteur.fr/SubtiList/). (TIF) [file pone.0066120.s003.tif]

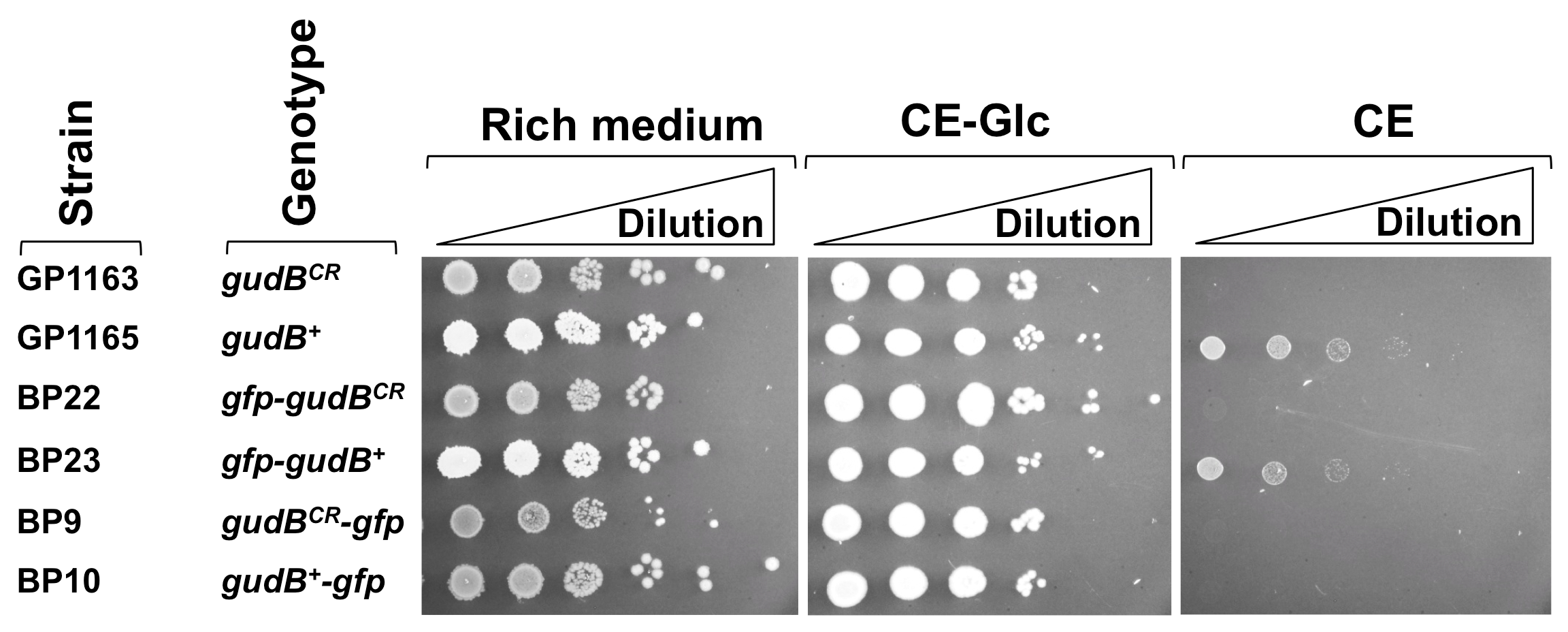

Supplement: Figure S4 — In vivo activities of GudBCR and GudB variants fused to GFP. 5 µl were plated from serial dilutions (from 10−1 till 10−6) of cell suspensions of the control strains GP1163 (rocG+ gudBCR) and GP1165 (rocG+ gudB+), and the strains BP22, BP23, BP9 and BP10 expressing the gfp-gudBCR, gfp-gudB+, gudBCR-gfp and gudB+-gfp fusions, respectively. The dilutions were spotted on SP medium agar plates (rich medium), and C minimal medium supplemented either with glucose and glutamate (CE-Glc medium) or with glutamate and ammonium (CE medium). The plates were incubated for 48 h at 37°C. (TIF) [file pone.0066120.s004.tif]

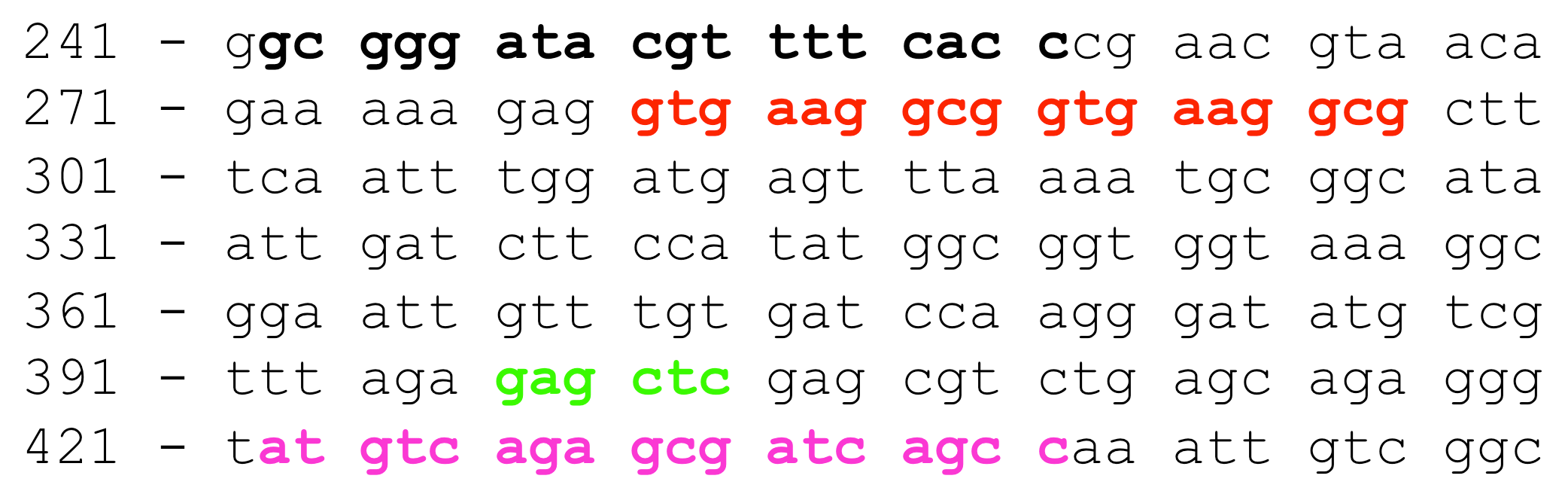

Supplement: Figure S5 — Part of the sequence of the gudBCRSac I -gfp allele. The recognition site GAGCTC for the restriction endonuclease SacI that is highlighted in green was generated by modification of the CTG leucine codon at position 402 to the leucine codon CTC (see Table S2). The 9 bp DR of the gudBCR gene is highlighted in red. Letters highlighted in black and pink indicate the regions where the oligonucleotides KG166 and KG196 hybridise. (TIF) [file pone.0066120.s005.tif]
