# Supplementary material for: Selection-Driven Accumulation of Suppressor Mutants in Bacillus subtilis: The Apparent High Mutation Frequency of the Cryptic gudB Gene and the Rapid Clonal Expansion of gudB+ Suppressors Are Due to Growth under Selection
Source: PLoS One. 2013 Jun 13;8(6):e66120. doi: 10.1371/journal.pone.0066120 (PMC3681913; doi:10.1371/journal.pone.0066120)
Supplement: Table S1 — Oligonucleotides. (DOCX) [file pone.0066120.s006.docx]

**Table S1. Oligonucleotides.**

| **Oligo** | **Purpose** | **Sequence^a^** |
| --- | --- | --- |
| FC159 | *dnaA*/tandem repeat | 5’-GCGGTCAAAGAAGATACATCTGATTTTCCTC |
| FC160 | *dnaA*/tandem repeat | 5’-CGCTACTGCGAGGGAAGCAGC |
| FC161 | *tcyA*/tandem repeat | 5’-GGAAGCGGCGAGGTTGTTGATC |
| FC162 | *tcyA*/tandem repeat | 5’-GCCAAGCGTCAATGCCGGCAG |
| FC163 | *yrvM*/tandem repeat | 5’-CAATGAATTAGCTATCGGAAAAGAAGGCC |
| FC164 | *yrvM*/tandem repeat | 5’-ggccgacgcctgaccgcg |
| FC165 | *aroH*/tandem repeat | 5’-ggcgattggaggagacatcatg |
| FC166 | *aroH*/tandem repeat | 5’-CTTCTTCAGTATCCCGTTCAACTGTAG |
| FC167 | *ykoW*/tandem repeat | 5’-GGCTGCGACCATTTGCAGGGC |
| FC168 | *ykoW*/tandem repeat | 5’-CGGCGCTGTTATTGCGACGGC |
| FC169 | *yrbF*/tandem repeat | 5’-GTTTGCGGTCCTTTACTTCTTGCTG |
| FC170 | *yrbF*/tandem repeat | 5’-CGTGCAATCCCCCGATTGTCACTAC |
| FC171 | *yisV*/tandem repeat | 5’-GAAGATCCATGTGTCACCGTCTGC |
| FC172 | *yisV*/tandem repeat | 5’-GAAGGTAGGACGGCTTCTCCGTC |
| FC173 | *yjzB*/tandem repeat | 5’-GAGAAGGAGGAGGAACAGCAAGAG |
| FC174 | *yjzB*/tandem repeat | 5’-CATAATTGAACCAATTTGGTCCATAATATGCATG |
| FC175 | *spoIIAA*/tandem repeat | 5’-GGACAGCTCGGGGCTTGGCG |
| FC176 | *spoIIAA*/tandem repeat | 5’-GCTTCACCGCAGGAGAGATAGCG |
| FC177 | *greA*/tandem repeat | 5’-GTAGATGAAGAAGTCACAGTACAAACACCG |
| FC178 | *greA*/tandem repeat | 5’-CACCCACATTGTGGACGAGGTGTTTC |
| FC179 | *amyE*/tandem repeat | 5’-GGTCTGATCGATGGGATGTCACGC |
| FC180 | *amyE*/tandem repeat | 5’-CCGTCTGCCCCGTCATTCAATGC |
| FC181 | *putP*/tandem repeat | 5’-GTCGATGTTCAGACGCTCAGCTTCAG |
| FC182 | *putP*/tandem repeat | 5’-CTCCCAAGCCAGAACCAACGCG |
| FC183 | *yhgE*/tandem repeat | 5’-GATCAGCTGCCTGACATCGCAACG |
| FC184 | *yhgE*/tandem repeat | 5’-CTCCCTCAGTTAATCTGTCAATCGCATC |
| FC185 | *rplL*/tandem repeat | 5’-CGGAGCTGCTGAAGAGCAAAGCG |
| FC186 | *rplL*/tandem repeat | 5’-CTTTAGCTTCTTTCAAGCCAAGACCAGTG |
| FC187 | *slrR*/tandem repeat | 5’-CAGCGGCGGTGAAGAAGAATGGC |
| FC188 | *slrR*/tandem repeat | 5’-GAGTCTGTTCGTAAAAGTGAACAATTCTTCC |
| KG92 | *gudB* gene | 5’-tttGGATCCtcattatatccagcctctaaaacgcg |
| KG118 | Sequencing | 5’-GCGGCTAAGAAGAGAGGCATCGATA |
| KG125 | *gudB* gene | 5’-AAAGGATCCgcaaaagttgtcggcatctcagatgC |
| KG126 | *gudB* gene | 5’-TTTGTCGACTATCCAGCCTCTAAAACGCGAAGCTTC |
| KG166 | *gudB*/tandem repeat | 5’-gcgggatacgttttcacc |
| KG167 | *gudB*/tandem repeat | 5’-caccgccatatggaagatc |
| KG180 | *gfp* gene | 5’-TTTGTATAGTTCATCCATGCCATGTGTAATC |
| KG181 | *gudB* gene | 5’-ACATGGCATGGATGAACTATACAAAATGGCAGCCGA  TCGAAACACCG |
| KG188 | *gudB* promoter | 5’-AAACAATTGCATTCAGCTTTCAGAAAGCTTACAGC  GAATC |
| KG189 | *gudB* promoter | 5’-TTTAGATCTGGATCCCCGGGAATTCgaatcttctgt  ttctcacatgctccctttc |
| KG190 | *gfp* gene | 5’-AAAGAATTCAA*AGGAGGA*AACAATCATGAGTAA  AGGAGAAGAACTTTTCACT |
| KG196 | *gudB* gene | 5’-ggctgatcgctctgacat |
| KG198 | *gudB* gene | 5‘[P]-gtcgtttagagagct**C**gagcgtctgagcag |
| KG199 | *cfp* gene | 5‘-AAACAATTGAA*AGGAGGA*AACAATCATGGTTTCAA  AAGGCGAAGAACTGTTTACG |
| KG201 | *yfp* gene | 5’-AAACAATTGAA*AGGAGGA*AACAATCATGGATTCAAT  AGAAAAGGTAAGCGAATTTGC |
| KG206 | *cfp* gene | 5’-TTTAGATCTtcattaCTTATAAAGTTCGTCCATGCC  AAGTGTAATG |
| KG208 | *yfp* gene | 5’-TTTAGATCTtcattaCTTGTACAGCTCGTCCATGCCGA |
| ST1 | *gudB* promoter | 5’AAAGAATTCCATTCAGCTTTCAGAAAGCTTACAGCGAATC |

**^a^)** Restriction sites are underlined. Mutations and ribosome binding sites are highlighted in bold and italic letters, respectively.
