# Supplementary material for: Selection-Driven Accumulation of Suppressor Mutants in Bacillus subtilis: The Apparent High Mutation Frequency of the Cryptic gudB Gene and the Rapid Clonal Expansion of gudB+ Suppressors Are Due to Growth under Selection
Source: PLoS One. 2013 Jun 13;8(6):e66120. doi: 10.1371/journal.pone.0066120 (PMC3681913; doi:10.1371/journal.pone.0066120)
Supplement: Table S2 — Plasmids. (DOCX) [file pone.0066120.s007.docx]

**Table S2. Plasmids.**

| **Plasmid** | **Purpose** | **Parent**  **plasmid** | **Construction (DNA, oligos, restriction enzymes)** | **Source** |
| --- | --- | --- | --- | --- |
| pAC5 | Integration of DNA into the *amyE* locus | - | **-** | [53] |
| pCFPbglS | Template for *cfp*  gene | - | - | [54] |
| pYFPbglS | Template for *yfp*  gene | - | - | [54] |
| pGP1870 | C terminal GFP  fusions from the native locus | - | - | [55] |
| pBP1 | C terminal GudB^CR^/GudB-GFP fusions | pGP1870 | *gudB^CR^,* KG125/KG126,  *Bam*HI/*Sal*I | This study |
| pBP7 | *gudB* promoter-driven gene expression from the *amyE* locus | pAC5 | *gudB* promoter, KG188/KG189,  *Mfe*I/*Bam*HI | This study |
| pBP8 | GFP-GudB^CR^ fusion | pBP7 | *gfp-gudB^CR^*, KG190/KG180 (*gfp*), KG181/KG92 (*gudB^CR^*),  *Eco*RI/*Bam*HI | This study |
| pBP9 | GFP-GudB fusion | pBP7 | *gfp-gudB* KG190/KG180 (*gfp*), KG181/KG92 (*gudB*),  *Eco*RI/*Bam*HI | This study |
| pBP11 | C terminal GudB*_Sac_*_I_^CR^-GFP fusion | pAC5 | *gudB^CR^_Sac_*_I_*-gfp*, ST1/KG198/KG197,  *Eco*RI/*Bam*HI | This study |
| pBP26 | *gudB* promoter-driven *yfp* gene expression from the *amyE* locus | pBP7 | *yfp*, KG201/KG208, *Mfe*I/*Bgl*II | This study |
| pBP27 | *gudB* promoter-driven *cfp* gene expression from the *amyE* locus | pBP7 | *cfp*, KG199/KG206, *Mfe*I/*Bgl*II | This study |
