# Supplementary material for: Selection-Driven Accumulation of Suppressor Mutants in Bacillus subtilis: The Apparent High Mutation Frequency of the Cryptic gudB Gene and the Rapid Clonal Expansion of gudB+ Suppressors Are Due to Growth under Selection
Source: PLoS One. 2013 Jun 13;8(6):e66120. doi: 10.1371/journal.pone.0066120 (PMC3681913; doi:10.1371/journal.pone.0066120)
Supplement: Table S3 — B. subtilis strains. (DOCX) [file pone.0066120.s008.docx]

**Table S3: *B. subtilis* strains.**

| **Strain** | **Genotype** | **Construction^a^** | **Source** |
| --- | --- | --- | --- |
| 168 | *trpC2 gudB^CR^* | - | laboratory collection |
| BP9 | *trpC2 amyE::(gltA-lacZ aphA3) ΔrocG::cat gudB^CR^-gfp spc* | pBP1🡪GP754 | This study |
| BP10 | *trpC2 amyE::(gltA-lacZ aphA3) ΔrocG::cat gudB-gfp spc* | pBP1🡪GP801 | This study |
| BP22 | *trpC2 ΔgudB^CR^::aphA3 rocG*::Tn*10 spc amyE::(gfp-gudB^CR^ cat)* | pBP8🡪GP1161 | This study |
| BP23 | *trpC2 ΔgudB^CR^::aphA3 rocG*::Tn*10 spc amyE::(gfp-gudB cat)* | pBP9🡪GP1161 | This study |
| BP31 | *trpC2 gudB^CR^ rocG::Tn10 spc amyE::(gudB^CR^_SacI_ gfp cat)* | pBP11🡪GP747 | This study |
| BP40 | *trpC2 gudB^CR^ amyE::(yfp cat)* | pBP26🡪168 | This study |
| BP41 | *trpC2 gudB^CR^ amyE::(cfp cat)* | pBP27🡪168 | This study |
| BP42 | *trpC2 gudB amyE::(gltA-lacZ aphA3) ΔrocG::cat* | The same as GP801 | This study |
| BP44 | *trpC2 gudB (T896G) amyE::(gltA-lacZ aphA3) ΔrocG::cat* | Derived from GP801 | This study |
| BP48 | *trpC2 gudB (Δ766) amyE::(gltA-lacZ aphA3) ΔrocG::cat* | Derived from GP801 | This study |
| BP46 | *trpC2 gudB (Δ673-738) amyE::(gltA-lacZ aphA3) ΔrocG::cat* | Derived from GP801 | This study |
| BP47 | *trpC2 gudB (C1222^ins^ T1223^ins^) amyE::(gltA-lacZ aphA3) ΔrocG::cat* | Derived from GP801 | This study |
| BP52 | *trpC2 gudB amyE::(cfp cat)* | BP41🡪GP804 | This study |
| BP156 | *trpC2 gudB amyE::(yfp cat)* | pBP26🡪GP804 | This study |
| GP342 | *trpC2 gudB^CR^ amyE::(gltA-lacZ aphA3)* | - | [18] |
| GP747 | *trpC2 gudB^CR^ rocG*::Tn*10 spc* | - | [20] |
| GP754 | *trpC2 gudB^CR^ amyE::(gltA-lacZ aphA3) ΔrocG::cat* | - | [22] |
| GP801 | *trpC2 gudB amyE::(gltA-lacZ aphA3) ΔrocG::cat* | - | [12] |
| GP804 | *trpC2 gudB amyE::(gltA-lacZ aphA3)* | - | [12] |
| GP1161 | *trpC2 ΔgudB^CR^::aphA3 rocG*::Tn*10 spc* | - | [27] |
| GP1163 | *trpC2 ΔgudB^CR^::aphA3 rocG*::Tn*10 spc amyE::(gudB^CR^ cat)* | - | [27] |
| GP1165 | *trpC2 ΔgudB^CR^::aphA3 rocG*::Tn*10 spc amyE::(gudB cat)* | - | This study |

**^a^)** Arrows indicate construction by transformation.
