# Supplementary material for: Selection-Driven Accumulation of Suppressor Mutants in Bacillus subtilis: The Apparent High Mutation Frequency of the Cryptic gudB Gene and the Rapid Clonal Expansion of gudB+ Suppressors Are Due to Growth under Selection
Source: PLoS One. 2013 Jun 13;8(6):e66120. doi: 10.1371/journal.pone.0066120 (PMC3681913; doi:10.1371/journal.pone.0066120)
Supplement: Table S4 — 9 bp long tandem repeats present in essential (indicated by a superscript “e”) and non-essential genes of the B. subtilis chromosome. (DOCX) [file pone.0066120.s009.docx]

**Table S4.** **9 bp long tandem repeats present in essential (indicated by a superscript “e”) and non-essential genes of the *B. subtilis* chromosome.** Tandem repeats can be either in frame or not in frame*.* The red letter indicates the nucleotide that renders the direct repeat imperfect. n. a., data not available.

| **Gene** | **Protein** | **Locus tag** | **Function** | **DNA sequence** | **Expression** [31] | |
| --- | --- | --- | --- | --- | --- | --- |
|  |  |  |  |  | Exp. phase (120 Min.) | Stat. phase  (400 Min.) |
| *gudB^CR^* | GudB^CR^ | BSU22960 | Inactive glutamate dehydrogenase | **gtg aag gcg gtg aag gcg** | Intermediate | Low |
| *putP* | PutP | BSU03220 | Proline permease | **ctg gct gtt ctg gct gtt** | Low | Intermediate |
| *greA* | GreA | BSU27320 | Transcription elongation factor | **gtg aaa att gtg aaa att** | High | Low |
| *spoIIAA* | SpoIIAA | BSU23470 | Anti-anti sigma factor | **aag caa att aag caa att** | Intermediate | High |
| *ykoW* | YkoW | BSU13420 | c-di-GMP synthase and phosphodiesterase | **gaa caa ttc gaa caa ttc** | Intermediate | Low |
| *amyE* | AmyE | BSU03040 | α-amylase | **aat aca caa aat aca caa** | Low | Intermediate |
| *aroH* | AroH | BSU22690 | Chorismate mutase | **att cgc gga att cgc gga** | High | Low |
| *yisV* | YisV | BSU10880 | Putative GntR family transcription factor | **gca ctt cag gca ctt cag** | Low | Low |
| *yjzB* | YjzB | BSU11320 | Unknown | **gtt tct cag gtt tct cag** | Low | Intermediate |
| *yhgE* | YhgE | BSU10160 | Unknown | **gcg cag gtg gcg cag gtg** | Intermediate | Low |
| *yrbF* | YrbF | BSU27700 | Unknown | **cag caa aag cag caa aag** | High | Low |
| *yrvM* | YrvM | BSU27540 | Unknown | **gga gtc ggg gga gtc ggg** | High | Low |
| *slrR* | SlrR | BSU34380 | Control of SlrA and SinR | **gtg caa gcc gta caa gcc** | Intermediate | High |
| *rplL^e^* | RplL | BSU01050 | Ribosomal protein L12 | **atc aaa gtt atc aaa gtt** | High | Intermediate |
| *tcyA* | TcyA | BSU03610 | Cysteine transporter binding protein | c**tt tct aaa att tct aaa a**aa | High | Low |
| *dnaA^e^* | DnaA | BSU00010 | Replication initiation protein | t**at act ttt gat act ttt g**tc | n. A. | n. A. |
