# Supplementary material for: Selection-Driven Accumulation of Suppressor Mutants in Bacillus subtilis: The Apparent High Mutation Frequency of the Cryptic gudB Gene and the Rapid Clonal Expansion of gudB+ Suppressors Are Due to Growth under Selection
Source: PLoS One. 2013 Jun 13;8(6):e66120. doi: 10.1371/journal.pone.0066120 (PMC3681913; doi:10.1371/journal.pone.0066120)
Supplement: Table S5 — Raw data of the experiment shown in Figure 4. (DOCX) [file pone.0066120.s010.docx]

**Table S5.** **Raw data of the experiment shown in Figure 4.**

| Time (h) | Experiment | Medium | *gudB^CR^* colonies (blue) | *gudB^+^* colonies (white) | *gudB^CR^* colonies (blue, in %) | *gudB^+^* colonies (white, in %) |
| --- | --- | --- | --- | --- | --- | --- |
| 0 | 1 | Preculture | 141 | 0 | 100 | 0 |
| 0 | 2 | Preculture | 73 | 0 | 100 | 0 |
| 0 | 3 | Preculture | 144 | 0 | 100 | 0 |
| 0 | 4 | Preculture | 156 | 0 | 100 | 0 |
| 7 | 1 | C-Glc | 167 | 0 | 100 | 0 |
| 7 | 2 | C-Glc | 219 | 0 | 100 | 0 |
| 7 | 3 | C-Glc | 145 | 0 | 100 | 0 |
| 7 | 4 | C-Glc | 184 | 0 | 100 | 0 |
| 7 | 1 | CE-Glc | 165 | 0 | 100 | 0 |
| 7 | 2 | CE-Glc | 129 | 0 | 100 | 0 |
| 7 | 3 | CE-Glc | 178 | 0 | 100 | 0 |
| 7 | 4 | CE-Glc | 104 | 0 | 100 | 0 |
| 7 | 1 | SP | 72 | 2 | 97,3 | 2,7 |
| 7 | 2 | SP | 91 | 4 | 95,8 | 4,2 |
| 7 | 3 | SP | 72 | 3 | 96 | 4 |
| 7 | 4 | SP | 48 | 0 | 100 | 0 |
| 24 | 1 | C-Glc | 173 | 0 | 100 | 0 |
| 24 | 2 | C-Glc | 127 | 1 | 99,2 | 0,8 |
| 24 | 3 | C-Glc | 180 | 0 | 100 | 0 |
| 24 | 4 | C-Glc | 208 | 1 | 99,5 | 0,5 |
| 24 | 1 | CE-Glc | 111 | 2 | 98,2 | 1,8 |
| 24 | 2 | CE-Glc | 86 | 6 | 93,5 | 6,5 |
| 24 | 3 | CE-Glc | 109 | 3 | 97,3 | 2,7 |
| 24 | 4 | CE-Glc | 121 | 24 | 83,4 | 16,6 |
| 24 | 1 | SP | 1 | 107 | 0,9 | 99,1 |
| 24 | 2 | SP | 16 | 105 | 13,2 | 86,8 |
| 24 | 3 | SP | 4 | 70 | 5,4 | 94,6 |
| 24 | 4 | SP | 9 | 171 | 5 | 95 |
